# Optimized breast cancer diagnosis using self-adaptive quantum metaheuristic feature selection

**DOI:** 10.1038/s41598-025-05014-z

**Published:** 2025-06-06

**Authors:** Alok Kumar Shukla, Shubhra Dwivedi, Deepak Singh, Sunil Kumar Singh, Diwakar Tripathi, Ram Kishan Dewangan

**Affiliations:** 1 Thapar Institute of Engineering and Technology, Patiala, Punjab, India; 2 National Institute of Technology, Raipur, India; 3 Department of Computer Science and Engineering, Manipal Institute of Technology Bengaluru, Manipal Academy of Higher Education, Manipal, India; 4 National Institute of Technology, Jamshedpur, India; 5 SCSET, Bennett University, Greater Noida, India

**Keywords:** Teaching Learning-based optimization, Breast Cancer, Feature selection, Genetic algorithm, Quantum, Support vector machine, Energy science and technology, Engineering, Mathematics and computing

## Abstract

Breast cancer is a leading cause of mortality among women and is increasing rapidly around the world. For early diagnosis of breast cancer, precise classification, and finding the best subset for cancer identification, evolutionary-based feature selection methods play a vital role in effective treatment. Previous studies have shown that existing evolutionary methods are complicated in correctly differentiating BC disease subtypes with high consistency, which seriously affects the performance of classification methods. To prevent diagnostic errors with hostile implications for patient health, in this study, we develop a new evolutionary method called SeQTLBOGA that incorporates the learner quantization before the search capability of the feature space to prevent premature falls into the local optima. In the SeQTLBOGA algorithm, quantum theory and a self-adaptive mechanism are employed to update the Teaching Learning-based Optimization (TLBO) rule to enhance convergence search capabilities. Most importantly, a self-adaptive genetic algorithm (GA) is also incorporated into TLBO to tradeoff between exploration and exploitation to handle slow convergence and exploitation competence, and simultaneously optimizing parameters of support vector machines (SVM) and the best features subset is our primary objective. Comparative results based on optimal computing time and performance are also offered to empirically analyze the traditional algorithms. Therefore, this paper aims to evaluate the most recent quantum-inspired metaheuristic algorithms in WBCD and WDBC databases, emphasizing their advantages and disadvantages.

## Introduction

Cancer is not brought on by external factors; rather, it is produced by specific genetic abnormalities in cells^1^. One of the biggest worries in the world, especially for women, is the rising number of cancer-related deaths^2^. This keeps up the pattern that breast cancer (BC) is the second most common cause of cancer-related deaths among women, and the cancer that is diagnosed in women more often than skin cancer. As of 2020^3^, lung cancer has been surpassed by breast cancer as the most frequent cancer worldwide. By 2040, there will likely be 3.2 million female cases of breast cancer (compared to the present 2.3 million instances), and 1 million projected deaths from the disease. The good news is that, if found early enough to prevent metastatic cancer, the 5-year survival rate for breast cancer can reach 90%^4,5^.

Therefore, figuring out how to efficiently extract critical features for breast cancer diagnosis and reduce dimensionality to ease computational and space complexity while enhancing model performance is crucial. Many automated procedures are available to differentiate between benign and malignant tumors^6,7^. Quantum computing (QC), one of the best ways to solve the dimensionality ruin problem, has been applied to enhance the efficiency of the automated process for early cancer diagnosis^8^. The concept of quantum computing is based on the principles of the quantum bit, promotes population variety because of the combination of states, and swift convergence^9^. Generally speaking, feature selection (FS) works by framing the task of finding a subset of characteristics as an optimization problem. For such an approach, a cost function that gauges a feature subset’s quality, usually via a classifier’s accuracy, must be defined^10^.

Recently, the principles of quantum computing have been applied to address the time and space complexity by combining natural methods such as artificial bee colonies, teaching learning-based optimization, particle swarm optimization (PSO), grasshopper optimization algorithm (GOA), GAs, and differential evolution^11^. Quantum evolutionary algorithms (QEAs) outperformed standard EAs for combinatorial optimization while avoiding premature convergence^12^. Furthermore^13^, frequently experience problems, including poor convergence accuracy and a tendency to converge towards the Local Optima (LO). The study presented an integrated strategy that integrates the techniques for Differential Evolution, Slime Mold Algorithm, and Salp Swarm Algorithm to address these issues presented by the SDSSA—this cutting-edge hybrid MIS model combined components from the SSA, SMA, and DE algorithms. The SDSSA model primarily depended on 2D Kapur’s entropy and a non-local means 2D histogram. It was first contrasted with analogous algorithms to assess the suggested approach efficiently.

The approach to decision-making in many aspects of life has been revolutionized by the ongoing development of artificial intelligence (AI). A growing area of artificial intelligence called Explainable Artificial Intelligence focuses on creating a wide range of devices and methods for opening up BlackBox AI solutions by producing transparent, perceptive, and understandable explanations of AI judgments^14^. Moreover, medical practitioners’ confidence in AI systems in the future may be strengthened by explainability in AI. It is not so much a product of AI as it is a challenge that has existed for at least as long. In^15^, the author presented a novel approach that merges a lightweight parallel depth-wise separable convolutional neural network (LPDCNN) with a ridge regression extreme learning machine to precisely classify three lung cancer types alongside normal lung tissue using CT images. The proposed methodology combined contrast-limited adaptive histogram equalization and Gaussian blur to enhance image quality, reduce noise, and improve visual clarity. LPDCNN extracted discriminant features while minimizing computational complexity (0.53 million parameters and 9 layers). The ridge-ELM model was developed to enhance classification performance, replacing the traditional pseudoinverse in the ELM approach. Notably, integrating the SHAP (Shapley Additive Explanations) in the proposed framework enhances Explainability, providing insights into decision-making and boosting confidence in real-world lung cancer diagnoses.

When tackling feature selection optimization issues, metaheuristic algorithms may typically meet two fundamental preconditions: exploration and exploitation, although they employ various operators and techniques^16^. The algorithm can further refine the quality of the result by searching inside a favorable region through exploitation, a local search capability. The best solution will be of higher quality if the two capabilities are balanced appropriately. Rao et al. proposed TLBO robust and relatively recent metaheuristic algorithm inspired by the dynamical behavior of teaching and learning events, to illustrate better the benefits and contributions of metaheuristics^17,18^. The drawbacks above can be addressed by genetic algorithms with self-adaptive parameters comprising crossover and mutation operators compared to other EAs. The quantity of a solution that can be obtained in terms of convergence speed is determined mainly by genetic algorithms’ crossover and mutation probabilities. Rather than relying on constant Pc and Pm values, SA-GA uses population data from every generation to modify Pc and Pm in an adaptive manner that preserves population variety and convergence ability.

Improving prediction skills mostly depends on choosing the most crucial variables or input parameters. Adopting an adaptive EA method that incorporates the advantages of multiple techniques has significantly improved the learning model performance; in most cases, adaptive algorithms outperform standalone algorithms. In real-world engine building, as opposed to other disciplines like feature engineering, there is a trade-off between feature dimension and the necessary computing cost, and the available feature space, or feature candidates, is typically constrained but noisy. The primary contributions of this paper are:

- This paper introduces quantum self-adaptive TLBO and GA algorithms, SeQTLBOGA, to determine the most useful features for the hybrid engine using SVM of BC in order to handle the stated technical obstacles methodically. In addition, the proposed method is applied to enhance the penalty factor and kernel parameter of the SVM.
- In order to improve exploration and exploitation search capability, the proposed algorithm first uses a quantum technique to generate solutions. After that, novel crossover and mutation operators are used to identify nearly optimal solutions and generate a population with incredible diversity.
- To provide interpretability, the prediction of the model can be interpreted through SHAP, which provides interpretable explanations.
- SeQTLBOGA aims to create a parsimonious predictive model that can distinguish benign from malignant breast tumors with the best features of high quality. In addition, this model can help the analyst provide diagnostic recommendations to general practitioners while achieving the desired prediction accuracy level.

The remainder of this study is organized as follows: Section “Related work” discovers related work on human intelligence-based algorithms and nature-inspired techniques. Section “Existing works” describes the data preparation and existing methods adopted for the study. Sections “Proposed methodology for classification system” and “Experimental study and analysis” describe the proposed work, and the results of the experiment are discussed in detail, followed by the concluding remarks in Section “Conclusion”.

## Related work

Healthcare professionals are currently gathering a wide range of patient data, especially in BC. In this regard, several EA-based decision-support systems have already received approval for use in clinical settings^19^. Wrapper techniques are still not widely used in clinical practice due to their interpretability, the ability to grasp how the algorithms make decisions, despite all the efforts to integrate them into physicians’ workflows. Therefore, given the previously indicated increase in BC data, rigorous examination of stochastic EAs should reveal pertinent insights on the impact of these techniques on the breast cancer subtyping problem. Since variations of EAs can learn embedded representations, we concentrate our study on self-adaptive and quantum EAs and their conditional equivalents^20^. Accurately identifying and describing the morphological traits is crucial to recognizing and diagnosing breast cancer disorders. The complicated morphological features restrict the success of human specialists in the field. Subjectivity and other human factors can lead to differences in diagnosis and treatment plans, which can harm patient outcomes. Practitioners could not be exposed to a variety of collections of breast cancer tissue samples, which can result in an incorrect diagnosis, such as false-positive or false-negative. Additionally, the manual procedure of assessing slides can take a long time, delaying the detection and management of the disease^21,22^.

Various sensing modalities can yield distinct tissue behavior for cancer and adjacent normal tissues, serving as a basis for cancer diagnosis. A novel multimodal diagnostic tool that can concurrently assess the optical, electrical, and mechanical bulk tissue properties can substantially augment the clinical findings, such as histopathology, potentially aiding the clinician in establishing an accurate and rapid cancer diagnosis. This review aims to discuss the clinical and engineering aspects and the unmet challenges of these physical sensing modalities, primarily in optical, electrical, and mechanical fields. The challenges of combining two or more of these sensing modalities that can significantly enhance the effectiveness of the clinical diagnostic tools are further investigated^23^.

A hybrid AlexNet-Extreme Learning Machine (ELM) approach for breast cancer diagnosis using mammography images was proposed in^24^. Batch normalization was applied to improve AlexNet’s performance, and the chimp optimization algorithm (ChOA) was utilized to avoid sub-optimal solutions in ELM. The Nelder-Mead simplex (NEMS) technique was then employed to enhance the convergence behavior of ChOA. The study’s main contributions were the proposed hybrid model and the application of ChOA and NEMS techniques to improve the performance of ELM. The proposed model was evaluated using the CBIS-DDSM dataset with Wiener and CALHE filters used as preprocessors. The effectiveness of the classification was examined using five optimization algorithms and several metrics. The outcomes demonstrated that the CALHE filter offered the best performance overall, and AlexNet-BN-ELM-CHOA-NEMS was the most accurate of the five models, with a sensitivity of 96.03%, specificity of 94.60%, and overall accuracy of 95.32%. The findings demonstrate the effectiveness of the proposed model in breast cancer diagnosis.

Breast cancer diagnosis involves the assessment of histopathological slide images by skilled pathologists. In^25^. The author was prone to human subjectivity, which can lead to diagnostic errors with adverse implications for patient health and welfare. Artificial intelligence-based models have yielded promising results in other medical tasks and offer tools for potentially addressing the shortcomings of traditional medical image analysis. The BreakHis breast cancer dataset suffers from insufficient data for the minority class with an imbalance ratio of more than 0.40, which poses challenges for deep learning models. To avoid performance degradation, researchers have explored a variety of data augmentation schemes to generate adequate samples for analysis. The author studied a Deep Convolutional Neural Network (DCGAN) with a specific generator and discriminator architecture to mitigate model instability and generate high-quality synthetic data for the minority class. The balanced dataset was passed to the fine-tuned ResNet50 model for breast tumor detection. The study produced high accuracy in diagnosing benign/malignant at 40X magnification, outperforming the state-of-the-art. The results demonstrated that deep learning methods can potentially support effective screening in clinical practice.

Malignant cells predominantly affect women, and researchers in the field of clinical sciences are increasingly interested in leveraging Artificial Intelligence techniques to predict and diagnose breast cancer. In^26^, the study utilized the widely-used Wisconsin Breast Cancer Dataset (WBCD) from the University of California, Irvine (UCI) machine learning repository. The dataset contains 30 features, including mean, standard error, and worst values, providing insights into different aspects of breast cancer. A stacked ensemble classifier was proposed to evaluate the effectiveness of the proposed model, incorporating multiple machine learning algorithms such as the Decision Tree Classifier, AdaBoost Classifier, Gaussian Naive Bayes, and Multi-layer Perceptron Classifier. Various performance measures, including Receiver Operating Characteristic Curve, Area Under the Curve, specificity, F1-score, sensitivity, and accuracy, were used to assess the model performance. The results demonstrated that the proposed ensemble technique achieved an accuracy of 97.66%, surpassing the performance reported in the existing literature. The study underscores the potential of AI techniques and machine learning algorithms in advancing breast cancer prediction and diagnosis, offering promising avenues for improving clinical decision-making and patient outcomes.

The shuffling frog leaping algorithm was combined with an innovative and effective minimum attribute reduction approach based on quantum-inspired self-adaptive cooperative co-evolution^27^. Initially, multi-state quantum bits were utilized to represent evolutionary frog individuals. Then, the self-adaptive quantum rotation angle and quantum mutation probability technique were implemented to update the quantum revolving door’s function. Second, a self-adaptive cooperative co-evolutionary model for minimal attribute reduction was created to partition the evolutionary attribute sets into manageable subsets. Based on their past performance records, the subsets were given the self-adaptive mechanism, and each one was evolved using the quantum-inspired shuffling frog leaping algorithm. Ultimately, the suggested algorithm’s global convergence was demonstrated theoretically, and its effectiveness was examined using UCI datasets, magnetic resonance, and a few other global optimization functions.

The Butterfly optimisation algorithm and the Ant Lion optimiser served as the foundation for the hybrid BOAALO approach, a feature selection technique that was developed in^28^. Three classifiers—an artificial neural network, a support vector machine, and an adaptive neuro-fuzzy inference system—were fed the optimal set of features selected by BOAALO in order to determine if breast tissue is benign or malignant. 651 mammography pictures were used to evaluate the suggested technique’s efficacy. The findings demonstrate that the first BOA and ALO are outperformed by the receiver operating characteristics curve, Type-I and Type-II error, kappa value, and BOAALO. Additionally, using a benchmark dataset, the suggested strategy’s robustness was evaluated and contrasted with five established methods.

Author^29^ developed a noninvasive artificial intelligence approach to characterize the tumor microenvironment by fusing deep learning’s prediction capabilities with the explainability of human-interpretable imaging phenotypes (IMPs). IMPs were created using 342 breast tumors’ 4D dynamic imaging linked to clinical and genomic data. This allows for the genetic relationship between cancer phenotypes and genotypes. A deep graph clustering technique with unsupervised dual attention was developed to divide bulk tumors into many geographically separated and phenotypically consistent subclusters. Intratumorous subcluster architecture, interaction, and proximity were captured using IMPs varying in spatial and kinetic heterogeneity. IMPs positively correlate with established TME markers and may accurately, consistently, and reliably predict specific molecular fingerprints, such as the expression of hormone receptors, epithelial growth factor receptors, and immunological checkpoint proteins.

## Existing works

High-dimensional datasets may require extensive computations to explore the space of possible search strategies fully^30,31^. For example, GA simultaneously handled feature and instance selection. The primary drive behind the development of quantum self-adaptive EAs is their efficacy in resolving medical issues. It is to be expected that the behavior of nature is always at its best. Probabilistic algorithms underpin all EA-based algorithms, and standard parameters like population size, tuning parameters, and maximum number of iterations are necessary. Many algorithms need their algorithm-specific control parameters and standard control parameters^32^. Utilizing the competition technique, a more recent study aims to efficiently search for less expensive features by enhancing the global search capability of an EA-based filter method for cost-sensitive feature selection.

### Quantum concept

The quantum concept denotes that specific physical attributes, such as energy, charge, and angular momentum, exist in discrete, indivisible units known as quanta, rather than in a continuous form. This essential concept underlies the entire domain of quantum mechanics, which elucidates the behaviour of matter and energy at atomic and subatomic levels. Quantum systems can simultaneously occupy several states until observation occurs. This underpins phenomena such as Schrödinger’s cat thought experiment, when a system exists in a superposition of potential outcomes until an observation occurs^33^. Each solution (feature subset) is represented as a qubit in superposition, enabling parallel evaluation of multiple states.

$$\:|\psi\rangle=\alpha\:|0\rangle+\beta\:|1\rangle,\:\text{where\:}|\alpha\:{|}^{2}+|\beta\:{|}^{2}=1$$

A rotation gate $\:U\left(\theta\:\right)$ adjusts qubit amplitudes to steer solutions toward high-fitness regions. For the BC problem, the quantum rotation gate is described as follows:

$$\:U\left(\theta\:\right)=\left[\begin{array}{cc}cos\theta\:&\:-sin\theta\:\\\:sin\theta\:&\:cos\theta\:\end{array}\right]$$

Applied to a qubit $\:|\psi\:\rangle$, the updated state becomes

$$\:|{\psi\:}^{{\prime\:}}\rangle=U(\theta\:)\cdot\:|\psi\:\rangle=\left[\begin{array}{c}\alpha\:cos\theta\:-\beta\:sin\theta\:\\\:\alpha\:sin\theta\:+\beta\:cos\theta\:\end{array}\right].$$

The rotation angle $\:\theta\:$ is dynamically tuned using fitness feedback. Large $\:\theta\:\:value$ promotes exploration by amplifying low-probability states. And, small$\:\:\theta\:\:value\:$enhances exploitation near high-fitness solutions. Generate $\:Q\left(t\right)$ quantum population with qubits in equal superposition. Collapse qubits into classical solutions via probabilistic sampling.

$$\:{\:x}_{ij}=\left\{\begin{array}{lll}1&\:\text{if\:}|{\beta\:}_{ij}{|}^{2}&\:\text{rand}\left(\text{0,1}\right)\\\:0&\:&\:\text{otherwise}\end{array}\right.$$

Compute $\:f\left({x}_{i}\right)$ for all solutions. Adjust $\:{\alpha\:}_{ij},{\beta\:}_{ij}$ using $\:U\left({\theta\:}_{ij}\right)$ to steer solutions toward better candidates. For an $\:n$-dimensional feature selection problem, quantum population $\:Q\left(t\right)\{{q}_{1}^{t},{q}_{2}^{t},\dots\:,{q}_{m}^{t}\}$ is maintained, where each $\:{q}_{i}^{t}$ is a matrix of qubits:$$\:{q}_{i}^{t}=\left[\begin{array}{cc}{\alpha\:}_{i1}&\:{\beta\:}_{i1}\\\:{\alpha\:}_{i2}&\:{\beta\:}_{i2}\\\:\vdots&\:\vdots\\\:{\alpha\:}_{in}&\:{\beta\:}_{in}\end{array}\right]$$

Here, $\:{Q}_{\left(t\right)}=\{\:{q}_{1}^{t}\:,{q}_{2}^{t}\:,\:{q}_{3}^{t},\:\dots\:,{q}_{N}^{t}\:\}\:$ is a population of N qubit learners at generation t; $\:{Q}_{\left(t\right)}\:$is ith 1, 2,…, N, individual defined as above. Each qubit’s superposition allows simultaneous exploration of $\:{2}^{n}$ Feature combinations exponentially increase search space coverage compared to classical methods.

Quantum superposition and adaptive rotation angles reduce the risk of stagnation in local optima. Gate entangle qubits to ensure correlated feature updates, enhancing global search coherence. In this way, the n-qubits learner can simultaneously represent the information of 2^n^ binary states. QEAs have better characteristics of diversity than classical approaches since they can represent a linear superposition of many states, thanks to the qubit representation. For the update process of QEAs, a suitable quantum gate $\:U\left(\theta\:\right).$ it is usually adopted in compliance with the BC problem.

$$\:{q}_{i}^{t}=\:\left|\begin{array}{cc}{{\alpha\:}_{1}^{t}}_{1}&\:.\dots\:\dots\:{{\alpha\:}_{i}^{t}}_{m}\\\:{{\beta\:}_{1}^{t}}_{1}&\:\dots\:\dots\:.{{\beta\:}_{i}^{t}}_{m}\end{array}\right|$$

and $\:{P}_{\left(t\right)}=\{\:{X}_{1}^{t}\:,{X}_{2}^{t}\:,\:{X}_{3}^{t},\:\dots\:,{X}_{N}^{t}\:\}\:$ is a set of binary solutions of observation states of Q(t), where $\:{X}_{i}^{t}$ is the binary solution by observing $\:{q}_{i\:}^{t}(\text{1,2}\dots\:n)$. In the initialize Q (t)’ step, each pair of qubit probability amplitudes, at $\:{\alpha\:}_{1r}^{t}\:$and $\:{\beta\:}_{1r}^{t}$; *r* = 1,2,…, m, are initialized with $\:\frac{1}{\surd\:2}$, $\:\forall\:{q}_{i\:}^{t}\in\:\text{Q}\left(\text{t}\right).\:$The next step generates a set of binary solutions $\:{P}_{\left(t\right)}=\{\:{X}_{1}^{t}\:,{X}_{2}^{t}\:,\:{X}_{3}^{t},\:\dots\:,{X}_{N}^{t}\:\}\:$where each bit of $\:{X}_{i}^{t}=\text{1,2},\dots\:,N$, is formed by determining the explicit state of each qubit of $\:{q}_{i\:}^{t}\:|0$> state or |1 > state, according to either $\:{\alpha\:}_{1r}^{t}\:$or $\:{\beta\:}_{1r}^{t}\:$of $\:{q}_{i\:}^{t}$. For example, to form an explicit state of rth bit of $\:{X}_{i}^{t}=\text{1,2},\dots\:,r$, the number r between 0 and 1 is generated randomly. The initial best solution $\:{X}_{i}^{t}$ best is then selected and stored among the binary solutions P (t). In the while loop, the quantum gate $\:U\:\left(\theta\:\right)$ is used to update Q (t-1) so that fitter states of the qubit chromosomes are generated. The jth qubit value $\:{\alpha\:}_{j}^{t}$; $\:{\beta\:}_{j}^{t}$ of $\:{q}_{i\:}^{t}$ is updated as below Eq. (1).

1 $$\:\left(\genfrac{}{}{0pt}{}{{\alpha\:}_{j}^{t+1}}{{\beta\:}_{j}^{t+1}}\right)=\:U\:\left({\theta\:}_{i}^{t}\:\right)\left(\genfrac{}{}{0pt}{}{{\alpha\:}_{j}^{t}}{{\beta\:}_{j}^{t}}\right)=\left[\begin{array}{cc}cos\:{\theta\:}_{j}^{t}\:&\:-\:sin\:{\theta\:}_{j}^{t}\:\\\:\text{sin}{\theta\:}_{j}^{t}\:&\:\text{cos}{\theta\:}_{j}^{t}\:\end{array}\right]\left(\genfrac{}{}{0pt}{}{{\alpha\:}_{j}^{t}}{{\beta\:}_{j}^{t}}\right).$$

Here, $\:{\theta\:}_{i}^{t}$ is the rotation angle of $\:{q}_{i\:}^{t}$. The best solution among P(t) is then selected in the next step, and if the current solution is fitter than the best-stored solution , the best-stored solution $\:{X}_{t}^{best}$will be replaced by this current solution.

### Teaching learning based optimization

Metaheuristic techniques incorporate procedures and strategies to create novel solutions and promote a proper balance of search diversification, intensification, and standard regulating parameters such as population size and maximum number of iterations^34^. It can take the form of two primary methods: population-based algorithms that evolve collections of solutions like TLBO and GA and single-solution-based algorithms that modify a single solution as SA. Because of the convergence rate and the reduced number of changing parameters, EAs are powerful. The algorithm known as Teaching Learning Based Optimization (TLBO) was created lately and is based on the chasing mechanism of the knowledge-acquisition phase, inspired by the classroom teaching and learning process. Owing to its distinct structure, TLBO possesses virtuous global search capabilities. The low convergence rate of the individual TLBO algorithm is one of its shortcomings, which restricts its practical use. When tackling complex global optimization issues, SA-GA smoothly enters the local optimal region while exhibiting a rapid rate of convergence, provided that sufficient fine-tuning of preliminary constraints is not required. An active SeQTLBOGA algorithm is suggested to solve the feature selection challenge given the features of TLBO and SA-GA.

This algorithm aims to identify the most significant learner by having students work together and share information. It is divided into two phases: the teacher and student phases. Students gain knowledge from their classmates and a capable teacher during both. For the TLBO algorithm to work, there can be very skilled students who can get excellent marks or test scores. Together, these students make up what is known as a class. In this class, information is shared through the neighbor learning phenomenon, which facilitates the sharing of perspectives and knowledge:

$$\:{X}_{i,k}=\{{X}_{i,1},\:{X}_{i,2},\:\dots\:,{X}_{i,Fe}\}$$

Where, $\:{L}_{b}\:is\:\text{l}\text{o}\text{w}\text{e}\text{r}\:\text{b}\text{o}\text{u}\text{n}\text{d}\:$and $\:{U}_{b}\:is\:\text{u}\text{p}\text{p}\text{e}\text{r}\:\text{b}\text{o}\text{u}\text{n}\text{d}\:$of the Fe dimension in the search space $\:{X}_{i,Fe}\in\:\:[\:{L}_{b}\:,{U}_{b}]$. The learner is randomly initialized within the search space capabilities. The evolution of $\:{X}_{i,k}$ is randomly generated by the following Eq. (2).

2 $$\:{X}_{i,k}={L}_{k}+\:{r}_{1}\:\text{*}\:({U}_{k}-\:{L}_{k}).$$

Where, i = 1, 2, 3,. . ., nop,$\:\:k=\text{1,2},3,\:\dots\:,Fe$, $\:{r}_{1}\:$shows a random variable, $\:{L}_{k}\:$ shows the lower bound and $\:{U}_{k}$ shows the upper bound value. The simulation of the classical learning process for all learners is categorized into two significant phases of the TLBO algorithm, namely the Teacher phase and the Learner Phase.

#### Teacher phase

This stage involves the teacher teaching the students. The instructor improves the mean grade for the class as a whole by keeping the class informed and interacting. The teacher is a gauge of how near the best outcomes from optimization problems have been achieved thus far. A skilled teacher refreshes their expertise based on the students’ understanding. The highest point to which a teacher can boost the class mean score will depend on the skill of the entire class. Assume that $\:{M}_{i,k}$= (1/nop) (Σ$\:{X}_{i,k}$) represents the average value for the given subject, with k = 1, 2,…K The process’s update equation, as outlined in Eq. 3.

3 $$\:{X}_{i,k}^{new}=\:{X}_{i,k}^{old}+\:{r}_{2}\text{*}({X}_{teacher,k}-\:{T}_{f}\text{*}{M}_{i,k})\&\:{T}_{f\:}=\:\text{r}\text{o}\text{u}\text{n}\text{d}\:[1+\text{r}\text{a}\text{n}\text{d}\:(0,\:1\left)\right]$$

Here, $\:{X}_{teacher,k}$ is the best learner of the adopted population at the current iteration of the algorithm, 2 is the random number lies between 0 and 1, respectively; $\:{T}_{f}$ is a teaching factor that decides the value of the mean to be changed. In every iteration, $\:{X}_{i,k}^{new}\:$is updated from the value of $\:{X}_{i,k}^{old}$. $\:{X}_{i,k}^{new}$ and $\:{X}_{i,k}^{old}$ denote the kth learners select after or before learning from the teacher^35^.

#### Learner phase

The second part of this process is called the learner phase, and it attempts to increase students’ knowledge in two ways: first, by having teachers provide feedback, and second, by having students engage with one another. To develop their communication abilities, each student’s goal is to interact with other students randomly^17^. To put it another way, every learner engages in interpersonal contact during the student phase. As a result, the surrounding solution may get trapped in a local optimum, leading to the current solution getting stuck in a local optimum close to the optimal solution. $\:{X}_{p}$ and $\:{X}_{q}$ where through mutual interaction, to choose the ith learner where (p$\ne$q), In the learning phase, the updating equation of the ith learner $\:{X}_{p}$ is as follows:

- **For** i = 1: nPop.
- Randomly choose two learners $\:{X}_{p}$ and $\:{X}_{q}$
- $$\:\:\:\:\:\mathbf{i}\mathbf{f}\:\left({f(X}_{p}\right)<\:f\left({X}_{q}\right))$$
- $$\:\:\:\:\:\:\:\:new{X}_{i}=old{X}_{i}+r\text{*}({X}_{p}-{\text{X}}_{\text{q}}).$$
- Else
- $$\:\:\:\:\:\:new{X}_{i}=old{X}_{i}+r\text{*}({X}_{q}-{X}_{p}).$$
- **End if**.
- **End for**.

Where, r is a uniformly distributed random number between 0 and 1, $\:{f(X}_{p})\:$ and $\:{f(X}_{q})\:$are the best solution of the learners $\:{X}_{p}$ and$\:\:{X}_{q}$, respectively.

### Cuckoo search

The Cuckoo Search (CS) algorithm was introduced in 2009 by Xin-She Yang and Suash Deb^36^ as an optimization method inspired by nature. The way that cuckoo birds breed served as the model for this optimization problem-solving technique. The strategy is predicated on the fact that cuckoos are known to deposit their eggs in other bird species’ nests. The CS algorithm’s main ideas and procedures are to set the initial population of potential solutions, which are referred to as nests in an algorithmic context. Since each nest indicates a possible fix for the optimization issue, Levy Flight: A Levy flight is when a flock of cuckoos walks randomly through the search space. Step sizes in Levy flights, a random walk, are determined by a probability distribution.

#### Levy flight

A Levy flight behavior is incorporated into Cuckoo Search to replicate the haphazard steps cuckoos take when looking for nests. A heavy-tailed probability density function, or Levy distribution, can be used to predict the step sizes in the Levy flight. The following represents the Levy flight in Eq. (4).

4 $$\:\text{l}\left(\text{t}\right)\:=\:\:\text{*}\:\text{L}\text{e}\text{v}\text{y}\left(\right).$$

Where l(t) is the step size at time t, λ is a scaling factor, and Levy(α) represents random samples from a Levy distribution with a specific parameter α.

**Algorithm 1:**
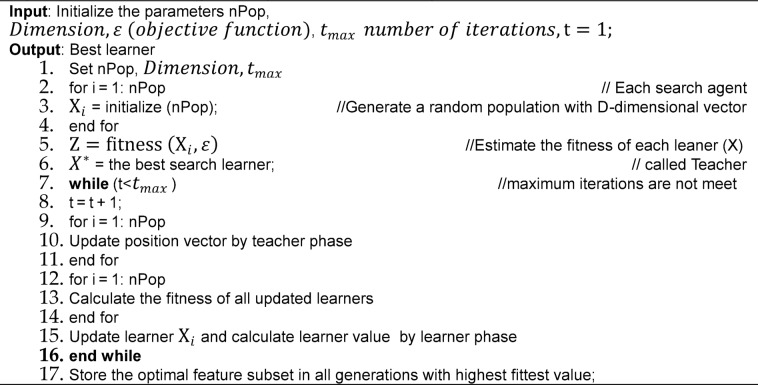
Basic Teaching Learning-based Optimization algorithm.

#### Egg laying and nest replacement

When one cuckoo’s egg is superior to what is currently in the nest, it replaces the previous one. Cuckoos lay their eggs in nests. The likelihood that a cuckoo egg will be superior to the contents of the nest is expressed in Eq. (5).

5 $$\:{P}_{a}=\:\text{e}\text{x}\text{p}(-{\upalpha\:}\:\text{*}\:\text{f}\text{i}\text{t}\text{n}\text{e}\text{s}\text{s}\_\text{c}\text{u}\text{c}\text{k}\text{o}\text{o}).$$

where fitness_cuckoo is the cuckoo egg’s fitness, $\:{P}_{a}$ is the acceptance probability, and α is a parameter governing the acceptance probability.

### Particle swarm optimization

Particle swarm optimization (PSO) is a kind of optimization that tackles a range of optimization problems by drawing inspiration from the natural world. PSO was first introduced by James Kennedy and Russell Eberhart in 1995^37^. PSO traverses the search space with a population of potential solutions, represented as particles, in order to find the optimal solution to an optimization issue. Every particle’s position in the search space represents a possible solution, and the particles travel based on their unique experiences as well as the collective behavior of the swarm. The PSO algorithm’s main ideas and procedures are as follows: Initialize the population of particles in the search space with random positions and velocities using the following update methods. Equations (6) and (7).

#### Position update

Using the particle current position, velocity, and best solution discovered thus far, update each particle’s position within the population. Equation (6) is the position update Eq. 

6 $$\:{x}_{i\:}(\text{t}\hspace{0.17em}+\hspace{0.17em}1)\:={x}_{i\:}\left(\text{t}\right)\:+\:{v}_{i\:}(\text{t}\hspace{0.17em}+\hspace{0.17em}1).$$

Where, $\:{x}_{i\:}$(t + 1) is the updated position of particle i at time t + 1, $\:{x}_{i\:}$(t) is the current position of particle i at time t, $\:{v}_{i\:}$(t + 1) is the velocity update of particle i at time t + 1.

#### Velocity update

The population best solution (gbest) and the best solution it has discovered thus far (pbest) Using the present velocity of each particle, determine its velocity. A possible solution to the optimization problem is represented by each particle. Determine the fitness (objective function value) of each particle’s current position to ascertain how effectively it solves the problem, Individual Best (best). Equation (7) is the velocity update Eq. 

7 $$\:{v}_{i\:}(\text{t}\hspace{0.17em}+\hspace{0.17em}1)\hspace{0.17em}=\hspace{0.17em}\text{w}\text{*}{v}_{i\:}\left(\text{t}\right)+\text{c}1\text{*}\text{r}\text{*}(\text{p}\text{b}\text{e}\text{s}\text{t}\_\text{i}-{x}_{i\:}\:(\text{t}\left)\right)+\text{c}2\text{*}\text{r}\text{a}\text{n}\text{d}\left(\right)\:\text{*}(\text{g}\text{b}\text{e}\text{s}\text{t}\:-{x}_{i\:}(\text{t}\left)\right).$$

Where, $\:{v}_{i\:}$(t + 1) is the updated velocity of particle i at time t + 1, $\:{v}_{i\:}$(t) is the current velocity of particle *i* at time t, *w* is inertia weight. c1 acceleration coefficients and c2 are control the influence of pbest and gbest, r is a random number between 0 and 1.

Based on each particle best-known position, update its individual best position. The global best position is updated based on identifying the particle with the best fitness among the total population. The pbest position is where the particle obtained the best fitness. Based on the particle’s current velocity, the difference between its current location and best, and the difference between its current position and best, update each particle’s velocity and position. According to the previously mentioned equations, the velocity update enables particles to move toward promising regions while exploring the search space. Fittest, gbest, velocity update, position update, and pbest iterations should all be repeated for a predetermined number of times, or until a stopping criterion is satisfied.

### Genetic algorithm

Genetic algorithm (GA) is the name given to an EA technique developed in 1975 that is modeled after natural selection^38^. It evolves a population of potential solutions across multiple generations to determine the optimal answer to a problem. The following provides a detailed description of each step in the genetic algorithm pseudo code of Algorithm 2 based on Darwin’s theory: At the start of the process, a population of individuals selected at random is initialized. Each chromosome represents a distinct strategy for solving the problem. The code in question consists of characters from a predefined character set into random phrases. Every fitness level of a chromosome is calculated. Here, fitness refers to a chromosome’s proximity to the desired result. Fitness is evaluated with the EAs by counting the characters that match between the desired sentence and the problem. Chromosomes are selected from the existing population to act as parents for the next generation. Fitness plays a part in selection; fitter people are more likely to be chosen. Candidates are selected with probability based on various selection techniques and fitness levels. To create offspring, selected parent pairs undergo crossover.

**Algorithm 2:**
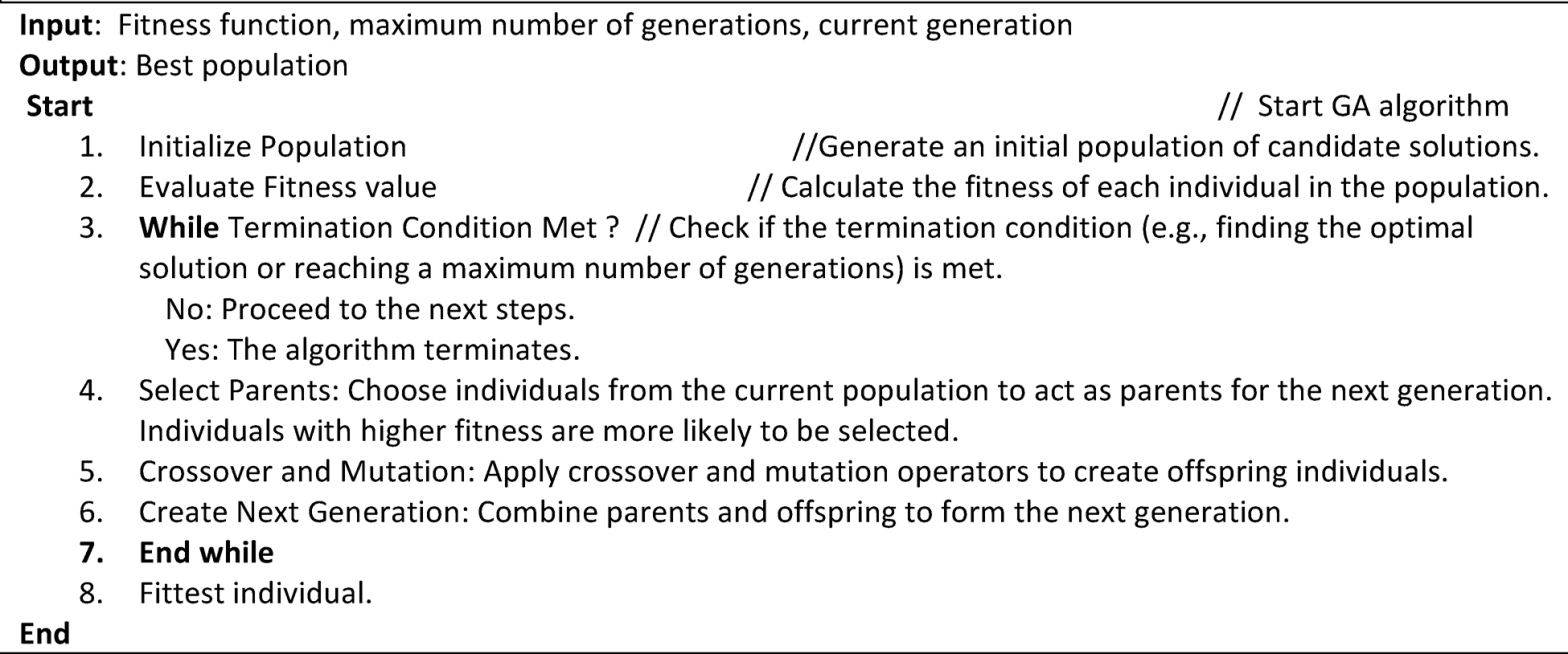
Pseudo code of Genetic algorithm.

#### Self-adaptive genetic algorithm

Genetic algorithms are robust optimization techniques, but usually, canonical ones concentrate on a limited number of parameters such as population size, number of crossovers, and mutation rate for each population. To improve the usability of GAs and increase efficiency in a dynamic environment, self-adaptation is an effective technique with several merits compared to canonical genetic algorithms, a difficult and time-consuming process^39^. This approach self-adapts to crossover and mutation-related parameters in other systems. We evaluate self-adaptive genetic algorithm performance on the BC type prediction issue against that of SVM, and the canonical genetic algorithm can be seen in Eqs. (8–9).

8 $$\:Pc=\left\{\begin{array}{c}\left[1-{c}_{1}\:\frac{\left(fmax-\:\text{f}\text{b}\right)}{\left(fmax-favg\right)}\:\right]-c2,\:\:fb\ge\:favg\\\:c3,\:\:\:\:\:\:\:\:\:\:\:\:\:\:\:\:\:\:\:\:\:\:\:\:\:\:\:\:\:\:\:\:\:\:\:\:\:\:\:\:\:\:\:\:\:fb<favg\end{array}\right.$$

9 $$\:Pm=\left\{\begin{array}{c}\left[1-{c}_{4}\:\frac{\left(\:\text{f}\text{b}\right)}{\left(fmax-fmin+favg\right)}\:\right]-c5,\:\:fb\ge\:favg\\\:c6,\:\:\:\:\:\:\:\:\:\:\:\:\:\:\:\:\:\:\:\:\:\:\:\:\:\:\:\:\:\:\:\:\:\:\:\:\:\:\:\:\:\:\:\:\:\:\:\:\:\:\:\:\:\:\:\:\:\:\:\:fb<favg\end{array}\right..$$

Here $\:fmax,\:fmin,\:favg,\:fb\:$represent the maximum value of the learner, the minimum value of the learner, the median value of the learner, and the top value after crossover and mutation in learners by the selection operation, respectively. c1 to c6 represent the constant value between 0 and 1. GA eliminates the requirement for domain experts to comprehend and adjust any operator-related parameter settings by self-adapting. We study how well it works to let EA make its parameter adjustments during a run, reducing the need for human parameter adjustment and improving the usability of EA for non-experts. Because of its self-adaptive methodology, the EA evolves many parameter values that specify the algorithm’s operation and potential solutions to a target problem. To cover a range of genetic operator behaviors, we incorporate several crossover and mutation operators in the list of possible operators. Algorithm 3 displays the SA-GA pseudocode.

**Algorithm 3:**
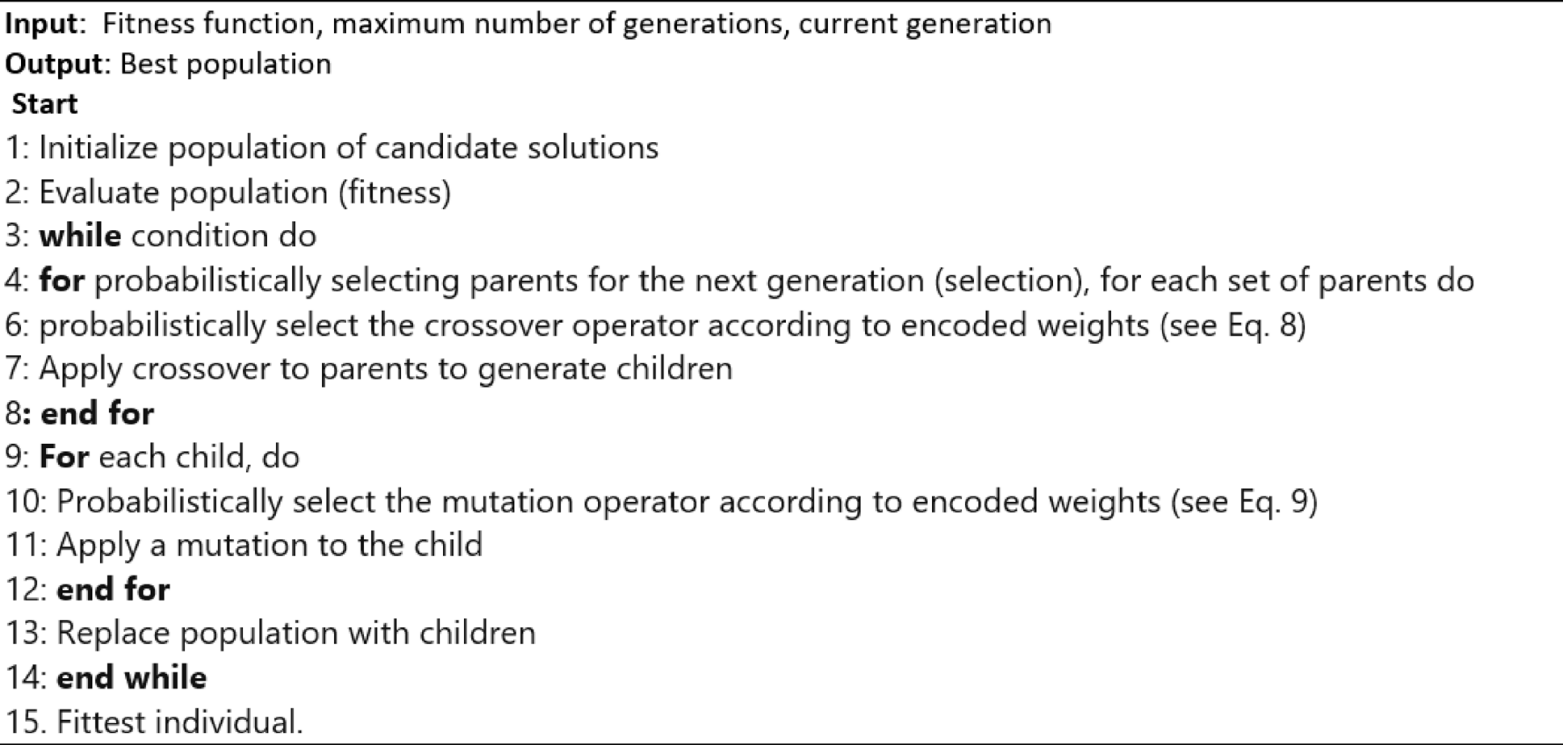
Pseudo-code of self-adaptive genetic algorithm (SA-GA).

## Proposed methodology for classification system

Previous attempts at developing self-adaptive-based metaheuristics have not successfully retained the appropriate feature sets to satisfy the various demands of practical medical systems. As a result, we recently formed a quantum self-adaptive TLBO with SA-GA, which is influenced by students’ intrinsic social intelligence and utilized to identify novel disease kinds. It also contains fewer control parameters, which reduces the cost of solving the FS issue in terms of convergence rate and computing time. Due to the population effect divergence, some individuals can quickly escape from the local optimum. However, most search learners descend into local optima, especially when the optimal solution also falls, so SeQTLBOGA, with SVM, can deteriorate.

### Quantum representation for feature selection

Feature selection aims to choose an optimal feature subset from all available features. Quantum representation for feature selection utilizes quantum computing techniques to improve the efficiency and efficacy of discovering pertinent features in BC datasets. It can simultaneously explore numerous feature combinations through quantum parallelism by encoding conventional data into quantum states, such as qubits or quantum superposition states. This facilitates a more rapid assessment of feature subsets in contrast to traditional methods, which frequently depend on iterative or greedy techniques.

Furthermore, quantum feature selection can leverage entanglement and interference to reveal correlations between features that classical methods may neglect. As a result, we reasoned for the representation of the features using qubits, with one qubit standing in for each feature. A series of qubits is used to represent every feature. If the state value of a qubit is |0 >, it indicates that the feature is selected, and if it is |1 >, it indicates that the feature is not selected. A string of qubits of length n, where n is the number of features, represents a population Qgi. Following is a representation of Qgi as follows:

$$\:{Q}_{gi}=\:\left|\begin{array}{cc}{\alpha\:}_{1}&\:\dots\:\dots\:\dots\:..{\alpha\:}_{n}\\\:{\beta\:}_{1}&\:\dots\:\dots\:\dots\:\dots\:{\beta\:}_{n}\end{array}\right|$$

|α|2 + |β|2 = 1, where |α|2 and |β|2 are the probabilities of states 0 and 1, respectively. Quantum population represents the superposition of the population positions, which contains 2n possibilities. The diversity of the population can be achieved with fewer individuals.

**Algorithm 4:**
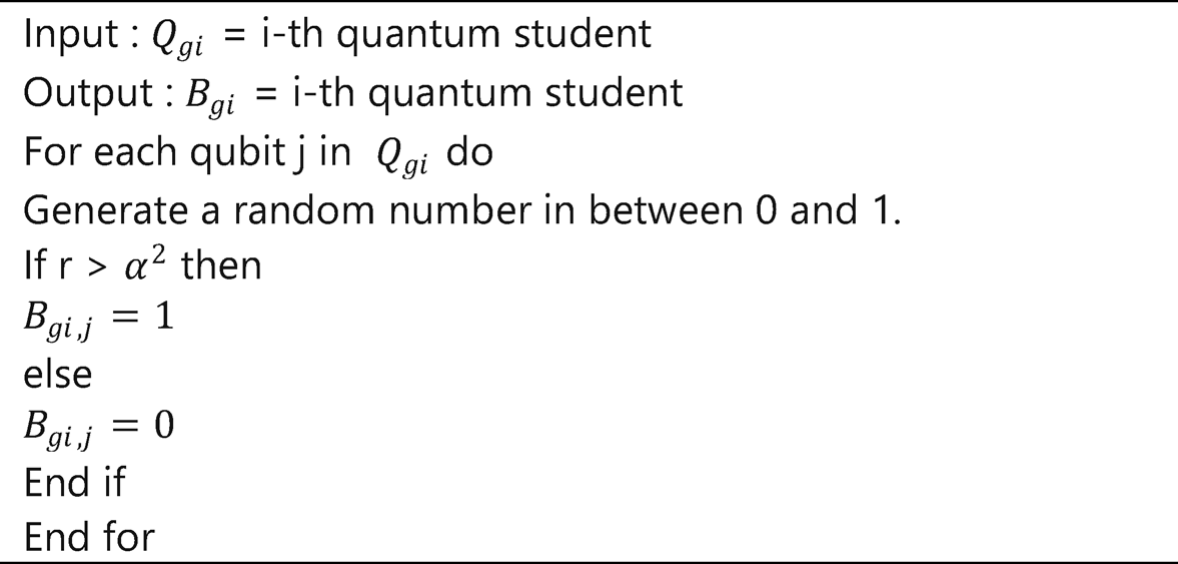
Pseudo code of quantum observation.

#### Quantum observation

The quantum student can through from quantum superposition to a definite state through quantum observation. A uniformly distributed random number r in the range [0, 1] is generated for each qubit. The relevant bit is assigned a value of 1 if r > sin2(θ), else a value of 0. Algorithm 3 displays an observation algorithm.

#### Distance between two binary learners

The SQTLBOGA method makes use of a Hamming distance version. In general, one can measure the difference between two symbol sequences using the Hamming distance. The number of unique bits between two binary answers in the BC problem is represented by the utilized distance. The proposed distance provides the ratio of the number of items not selected in the two binary students’ solutions to the Hamming distance.

- If d $$\:\left({B}_{gi,k},{B}_{gj,k}\:\:\right),\:where\:\:\:{B}_{gi,k}\:\ne\:\:{B}_{gj,k}$$ then
- $$\:{B}_{gi,k}=1$$
- else
- $$\:{B}_{gj,k}=0$$

$$\:\delta\:\:\left({B}_{gi}\:,{B}_{gj}\:\:\right)=\:\sum\:_{k=1}^{m}d\:({B}_{gi,k}\:,{B}_{gj,k}\:)$$

$$\:d\:\left({B}_{gi}\:,{B}_{gj}\:\right)=\:\frac{\delta\:\:\left({B}_{gi}\:,{B}_{gj}\:\:\right)}{2m-\left(\sum\:_{k=1}^{m}{B}_{gi,k}+\:\sum\:_{k=1}^{m}{B}_{gj,k}\:\:\right)}$$

where *m* is the size of a binary learner solution.

#### Quantum movement according to learner strategy

The probability amplitude state of quantum students is updated using a rotation gate; the update formula is presented below in Eq. (10).

10 $$\:\left(\genfrac{}{}{0pt}{}{{\alpha\:}^{d}(t+1)}{{\beta\:}^{d}(t+1)}\right)\:=\:\left[\begin{array}{cc}\text{c}\text{o}\text{s}(\varDelta\:{\theta\:}^{t})&\:-\text{sin}\left(\varDelta\:{\theta\:}^{t}\right)\\\:\text{sin}\left(\varDelta\:{\theta\:}^{t}\right)&\:\text{c}\text{o}\text{s}(\varDelta\:{\theta\:}^{t})\end{array}\right]\:\left(\genfrac{}{}{0pt}{}{{\alpha\:}^{d}\left(t\right)}{{\beta\:}^{d}\left(t\right)}\right).$$

$\:{\theta\:}_{i\:}^{t}=\:{\varDelta\:}_{d\:}^{i}\text{*}s\:\left({\alpha\:}_{d\:}^{i},\:{\beta\:}_{d\:}^{i}\:\right)$, represents the qubit rotation angle, where $\:{\alpha\:}_{d\:}^{i}$ is the size of rotation and $\:s\:\left({\alpha\:}_{d\:}^{i},\:{\beta\:}_{d\:}^{i}\:\right)$, is the direction of rotation which we can get from Table [56]. This strategy aids in maintaining a balance between early-stage exploration and late-stage exploitation. By leveraging historical information from the search process, the proposed approach can detect potential populations where the population becomes trapped in local optima. This enables the population to break free from the current local optima, enhancing its exploration capability.

### Weighted teaching learning-based optimization

TLBO method uses inertia weights assigned to distinct populations based on the rank of the population^44^ as demonstrated in the inertia weight factor, which observes the scenario to search and adjust the inertia weight value based on multiple feedback values in Eq. (11).

11 $$\:{{\upomega\:}\:}_{i\:\:\:\:\:\:}=\frac{{{Te}^{\text{*}}}_{i}}{{T}_{Pop}}\left(\:{{\upomega\:}}_{max}-\:{{\upomega\:}}_{min}\right)+{{\upomega\:}}_{min}.$$

Where $\:{{Te}^{\text{*}}}_{i}\:$is the rank of the i^th^ population when the population is ordered based on its population’s best fitness, $\:{{\upomega\:}}_{min}\:and\:{{\upomega\:}}_{max}$ is constant and $\:T\_Pop$ denotes as total number of populations.

#### Teacher updating phase

The metaheuristic approach known as teaching-learning-based optimization (TLBO) was motivated by the teaching and learning process in the classroom. The population is considered a group of pupils, and a fitness evaluation is used to choose the best student to be the teacher. The Teacher and Learner stages are the two distinct phases of the TLBO procedure. To avoid premature convergence, we use the weighted technique in TLBO to resolve problems of this kind. We seek to improve the learning represented in Eq. (12) using the wTLBO approach. This stage involves analyzing Eq. (2), knowing the precise solution (∗) and the person utilized in the subsequent iterations. Using the fundamental TLBO algorithm ($\:{Te}^{\text{*}}).\:$We generate the learners collectively from the previous position and best position; here, we introduced a new position-updating rule through an inertial weight strategy on basic TLBO, whereby the position of every class member is updated by the following Eq. (12). First, we randomly select learners for the full class. Second, we assess the best values of these two learners and get the best one.

12 $$\:{X}_{i,k}^{new}=\:{{\upomega\:}\:}_{i\:\:}\text{*}{X}_{i,k}^{old}+\:r\text{*}({X}_{{Te}^{\text{*}},\:k}-\:{T}_{f}\text{*}{M}_{i,k})\&\:{\text{T}}_{\text{f}}=\text{r}\text{o}\text{u}\text{n}\text{d}\:[1+\text{r}\text{a}\text{n}\text{d}\:(0,\:1\left)\right].$$

Where, $\:{X}_{i,k}^{new}$ and $\:{X}_{i,k}^{old}$ consist of the learner’s new value and old values, r is a random number in the range [0, 1], $\:{T}_{f}\:$is the teacher factor, with a range of^1,2^, random $\:{M}_{i,k}$ is created from the learner’s desired mean values, and $\:{Te}^{\text{*}}$ represents the top learner in the current iterations. For learners $\:p$ and $\:q$:$\:\:\left|{\psi\:}_{i}\right.\rangle\otimes\:\left|{\psi\:}_{q}\right.\rangle\stackrel{}{\to\:}\left|{\psi\:}_{p}\right.\rangle\otimes\:\left|{\psi\:}_{q}\oplus\:{\psi\:}_{p}\right.\rangle.\:$This ensures feature subset changes in one learner directly influence others, mimicking group learning. Instead of fixed $\:r$, crossover probabilities derive from qubit amplitudes ($\:{p}_{\text{cross}}=|\beta\:{|}^{2}$) promoting diversity. Mutation rates scale with qubit phase differences: ${p}_{m}\:\text{p}\text{r}\text{o}\text{p}\text{o}\text{s}\text{a}\text{n}\text{a}\text{l}\:\text{t}\text{o}\,{arccos}\left(\left|\langle{\psi\:}_{p}|{\psi\:}_{q}\rangle\right|\right).$This prevents stagnation in local optima, reducing feature redundancy. Quantum-evolved solutions undergo elitist selection and mutation:$\:\:{p}_{c},{p}_{m}$ over generations. This refines feature subsets while preserving quantum-enhanced diversity. By merging quantum superposition with the TLBO teaching paradigm, SeQTLBOGA achieves faster convergence and higher precision in BC classification, addressing TLBO’s historical limitations in feature selection stability.

**Fig. 1:**
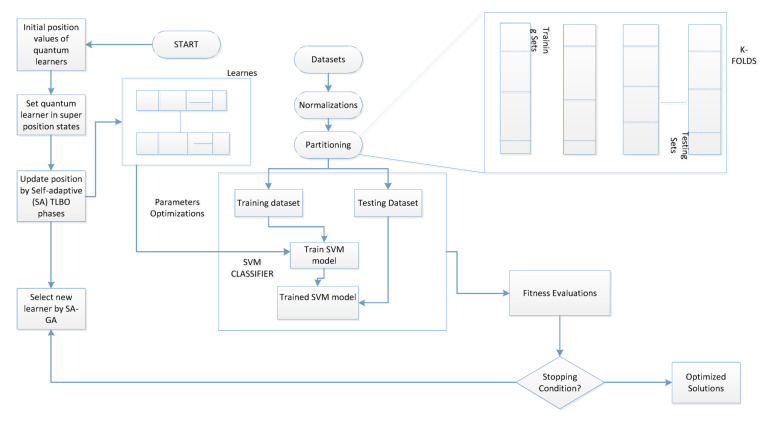
Optimizing parameters of SVM by the new optimizer SeQTLBOGA for breast cancer types detection.

The following advancements are incorporated into our process. To enable the system to use previous data or information from other patients, primary data must first be added to the records. Second, each action necessitates the algorithm to examine patient data, and a learner’s estimated knowledge can only be updated following the action. Third, our technique uses self-adaptive quantum TLBO and GA to minimise unnecessary characteristics from BC datasets. Creating a framework as shown in Fig. 1 is one possible solution for the aforementioned issues. The Boolean operator XOR (⨂) connects the best learner as the teacher and the learner. This is the first kind of work that uses self-adaptive (SA) processing with quantum TLBO and GA, which calls for many important advancements in comparison to the regular EAs learning works. Our framework uses SVM learning algorithms and feature selection that is inspired by nature to identify a patient’s illness early.

#### Self-adaptive teaching learning-based optimization

Studies conducted in the past have mainly concentrated on the self-adaptive (SA) processing of teaching elements. These factors are crucial for teachers because they indicate how much they impact students’ average performance. For instance, in 2020^40^, the author presented a hybrid adaptive teaching-learning-based optimization. Unlike in the original text, teaching factors are not a fixed value in this article. A strategy of adaptive selection can change its value.

$$[{T_f} = \left\{ {\begin{array}{*{20}{l}}{2,\:\:if\:\:RP}\quad{ <\in}\\{\:1,\:\:\:\:\:\:RP\:otherwise}&{}\end{array}} \right.$$

In the above formula, *RP* represents the learner’s ranking probability. This article establishes a threshold condition to determine the interim method for computing teaching factors. € is 0.5 as the threshold. Using the first technique, the teaching factor equals 2 when *RP* is greater than 0.5. If the second option is selected, the teaching factor remains at 1. In contrast to the ones mentioned before, the adaptive strategy in this study intends to process the instructional components adaptively by applying a new formula. The precise equation is as follows:

13 $$\:{T}_{f}=\text{cos}\left(\:\frac{i{\upomega\:}*it}{Maxit}\right).$$

The new teacher phase is defined by the following Eq. (14);

14 $$\:{X}_{i,k}^{new}=\:{{\upomega\:}\:}_{i}\text{*}{X}_{i,k}^{old}+\:r\text{*}({X}_{{Te}^{\text{*}},\:k}-\:{T}_{f}\text{*}{M}_{i,k}).$$

In the above Eqs. (13) and (14), *i*$\:{\upomega\:}$ and *it* refer to the inertia weight and current iteration, respectively. The *Maxit* refers to the maximum iteration. By this formula of Eq. (14), the teaching factor ($\:{T}_{f}$) adaptively changes. Other steps of self-adaptive quantum on TLBO with GA are the same as those of the original algorithm. The teacher and student levels updating process is still based on the original formulas (16). The SeQTLBOGA algorithm pseudo-code is given in Algorithm 5.

**Algorithm 5:**
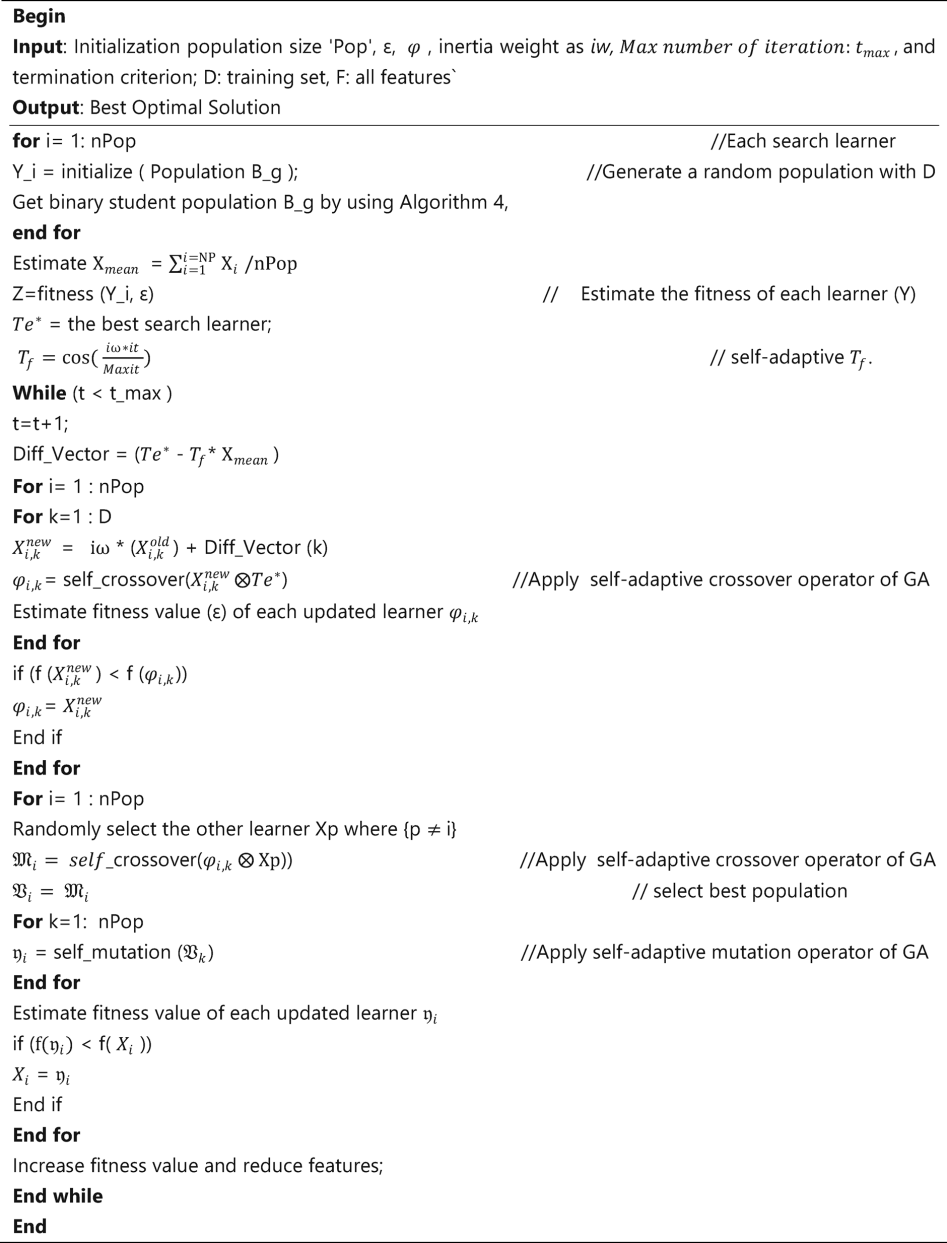
Pseudo-code SeQTLBOGA for feature selection.

In addition, evaluate the performance of SeQTLBOGA, we have used SVM as fitness function in the self –adaptive and quantum TLBO (see Fig. 1) and find the best agents with optimal number features.

The proposed processes for the optimized SVM model are as follows, as seen in Fig. 1. Set up the fundamental TLBO and GA settings. Give the students *n* + 2 dimensions to encode. Finally, there are two dimensions: C and σ. The values of the *n* dimensions are features, where 1 denotes that a feature is chosen and 0 denotes that a feature is discarded. Update the self-adaptive notion and apply the quantum concept to the teacher phase. Train the SVM and assess the learner fitness (FA) for every search. The ideal SVM parameters are obtained, and the learner fitness value is maintained if it approaches the best FA. A learner can get a high FA if they have a modest number of carefully chosen characteristics and a high classification accuracy (CA).

Furthermore, as the number of feature subsets is related to the SVM generalization error, the learner that scans fewer features obtains a more enormous FA. Thus, we consider each of them in this study and build the Fitness function as Eq. (15). To reach the FA, update learners through the self-adaptive genetic algorithm phase. Using the training dataset and the SVM optimal parameters, obtain the learning engine. To determine the best FA, use the model to predict test results. Obtain the feature subset, optimal C, and σ from the best learner (as Teacher).

**Fig. 2:**
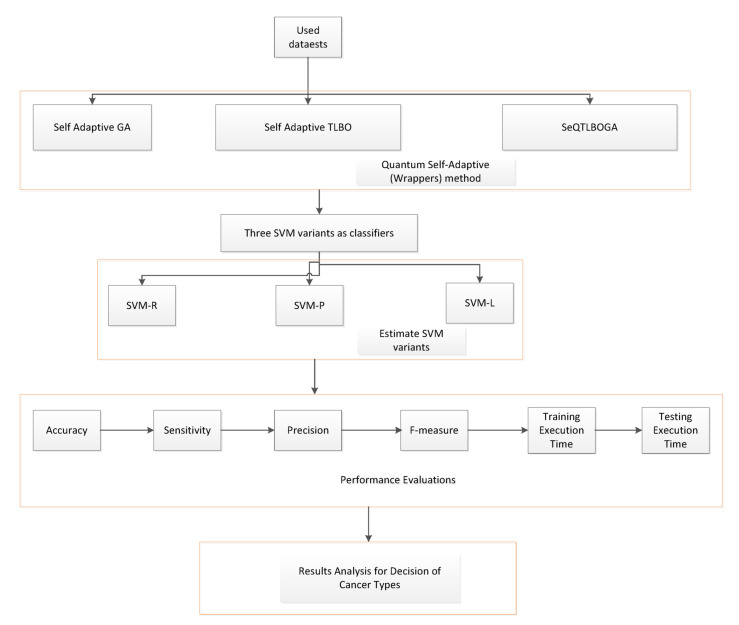
SeQTLBOGA implementation approach for prediction of BC disease.

### Optimizing parameters using proposed method

SeQTLBOGA, the most recent search technique used to identify the best answers to various real-world problems, is shown in Fig. 2 for predicting BC illnesses. It is a global search heuristic that mimics socially inspired behavior that humans influence. The first learner in TLBO is created arbitrarily using a set of candidate solutions, and it is then quantized using the idea of quantum computing^41^. In the instructor phase of the TF, an adaptive teaching factor takes the place of the original TLBO teaching factor. The algorithm’s overall performance is improved because the adaptive teaching factor can successfully balance search efficiency and convergence speed. After all potential learners have been assessed using fitness values, the best learner among the current learners is selected as the instructor. Other students begin to move in that direction as the group leader draws attention to themselves by drawing another person to the vicinity. GA searches the solution space by simulating survival of the fittest and using that evolution. To address the BC problem, they explore every region of the state space and exploit potential locations by applying adaptive behavior of crossover and mutation to individuals within the population. SeQTLBOGA finds kernel function parameters and feature vectors simultaneously. Learners must, therefore, possess two of these elements. In these experiments, we used the RBF kernel function for two reasons. First, the kernel can handle scenarios with a nonlinear relationship between the features and class labels because it places samples into a higher-dimensional space. The second explanation relates to the number of hyperparameters C and σ, which influence how complex the model is. The RBF kernel requires a maximum of two parameters.

Like other EAs, basic TLBO becomes locked in local optima when utilized with varying optimization challenges. As a result, the self-adaptive nature is combined with GA in TLBO, allowing the top learners to depart from local optima. Parameters σ and C of the SVM are optimized utilizing TLBO amalgamation of GA to combine the quantum search capability notion with the investigation of SeQTLBOGA. The process for SVM parameter optimization using SeQTLBOGA is divided into two parts, as shown in Fig. 1. a, SVM parameter encoding and population formation; b, fitting value computation for each learner based on Eq. (15). The calculation process involves the use of the testing dataset to classify whether or not diseases are present using an expert detection engine, the cross-validation of SVM model prediction, the application of the SeQTLBOGA model to train the SVM model for generating new solutions, and the selection of the best solution obtained by optimizing the SVM tuning parameter.

### Fitness function

The quality of each produced individual is assessed using predefined parameters. For this use, a fitness function should be defined as Eq. (15). Our model fitness function is determined by averaging the classification accuracy of SVM models trained using user-supplied data and parameters. The function of fitness, which is expressible, can be utilized to assess the quality of each individual $\:{l}_{i}^{t}$ at iteration t, where lab ($\:{x}_{j}$) is the classification result of the j^th^ instance in the testing data set, $\:{x}_{j}$ is the actual class label of the j^th^ instance, $\:\theta\:$ is the relation between c($\:{x}_{j}$), and $\:{y}_{j}$ that is if lab($\:{x}_{j}$) = $\:{y}_{j}$ then $\:\theta\:$ = 1 otherwise $\:\theta\:$ = 0. N is the number of instances in the testing data set. Note that the fitness access is measured based on k-cross-validation, where K denotes the number of folds. The latter step is essential to avoid the problem of overfitting and, consequently, to obtain more robust results. It can be referred as cross-validation to as an internal cross-validation

15 $$\:Fitnes{s}_{value\:}=\frac{1}{K}\:\sum\:_{k=1}^{N}\frac{1}{N}\:\sum\:_{J=1}^{N}\theta\:\:(lab\left({x}_{j}\:\right),{y}_{j}).$$

### Computational complexity

The computational complexity of SeQTLBOGA is expressed as $\:O\left(m\cdot\:n\cdot\:T\cdot\:{log}k\right)+$ O (T ∗ n) +(n ∗ m +¥).

Several key factors, including population size, dimensionality, chaos-inspired developments, self-adaptive strategies, and genetic algorithms, influence the computational complexity of the self-adaptive quantum Teaching Learning-based Optimization Algorithm, SeQTLBOGA. Understanding the complexity of SeQTLBOGA requires breaking down its significant computational steps and analyzing their impact on overall efficiency. Where, $\:m$: Population size, $\:n$: Number of features,$\:\:T$: Generations until convergence,$\:\:k$: Reduced feature subset size ($\:logk$ term from quantum mechanism) ,¥: Computation Training. SeQTLBOGA reduces time complexity compared to basic methods like GA, PSO, DE through wrapper-inspired and adaptive learning. For large-scale datasets, our quantum approach may complement its strengths.

The final computational complexity of our technique is expressed: $\:O\left(m\cdot\:n\cdot\:T\cdot\:{log}k\right)+$ O (T ∗ n) +(n ∗ m +¥)]. Suppose that $\:m$: Population size, $\:n$: Number of features,$\:T$: Generations until convergence,$\:k$: Reduced feature subset size ($\:logk$ term from quantum mechanism),¥: Computation Training. According to our algorithm, population initialization for computation training is ¥ times. It needs O (n ∗ $\:{log}k$) time complexity to transform qubit learners to binary learners. The position of each learner needs to be updated, which takes O($\:{log}k$) time complexity. An algorithm uses a Self-adaptive strategy to find a better solution, which takes time complexity O(n). Therefore, the overall time complexity of our algorithm is $\:O\left(m\cdot\:n\cdot\:T\cdot\:{log}k\right)+$ O (T ∗ n) + (n ∗ m +¥).

### Model interpretation

#### Explainable AI models

This section addresses the strategies and tactics that can be applied to improve the intuitiveness of machine-learning models for human comprehension. In the context of our method, explainability is the capacity to comprehend and analyze the choices or forecasts made by a model. This entails bringing the evolutionary system decision-making process into clear and human-understandable terms. Furthermore, it is essential to foster systemic trust, particularly in BC applications, where poor decisions can have grave repercussions. Several strategies for attaining explainability have been examined in previous research, including using interpretable models, producing explanations for intricate models, and creating models with naturally interpretable structures^42,43^. Developers can guarantee that the model is accurate but also dependable and trustworthy by integrating Explainable AI (XAI) approaches. Shapley Additive Explanations (SHAP) in the XAI technique evaluate the impact of individual attributes on machine learning model predictions^15^. The model baseline performance is assessed by comparing the data. Consequently, each feature is evaluated by permuting its value randomly, reassessing the model performance, and determining how it deviates from the baseline. A negative score suggests a potential dependency on other variables, whereas a more considerable performance decline denotes more significant elements. Additionally, the ability of our model to continue operating and producing accurate forecasts in the face of unanticipated or hostile conditions is referred to as the proposed method of resilience in this context.

**Fig. 3:**
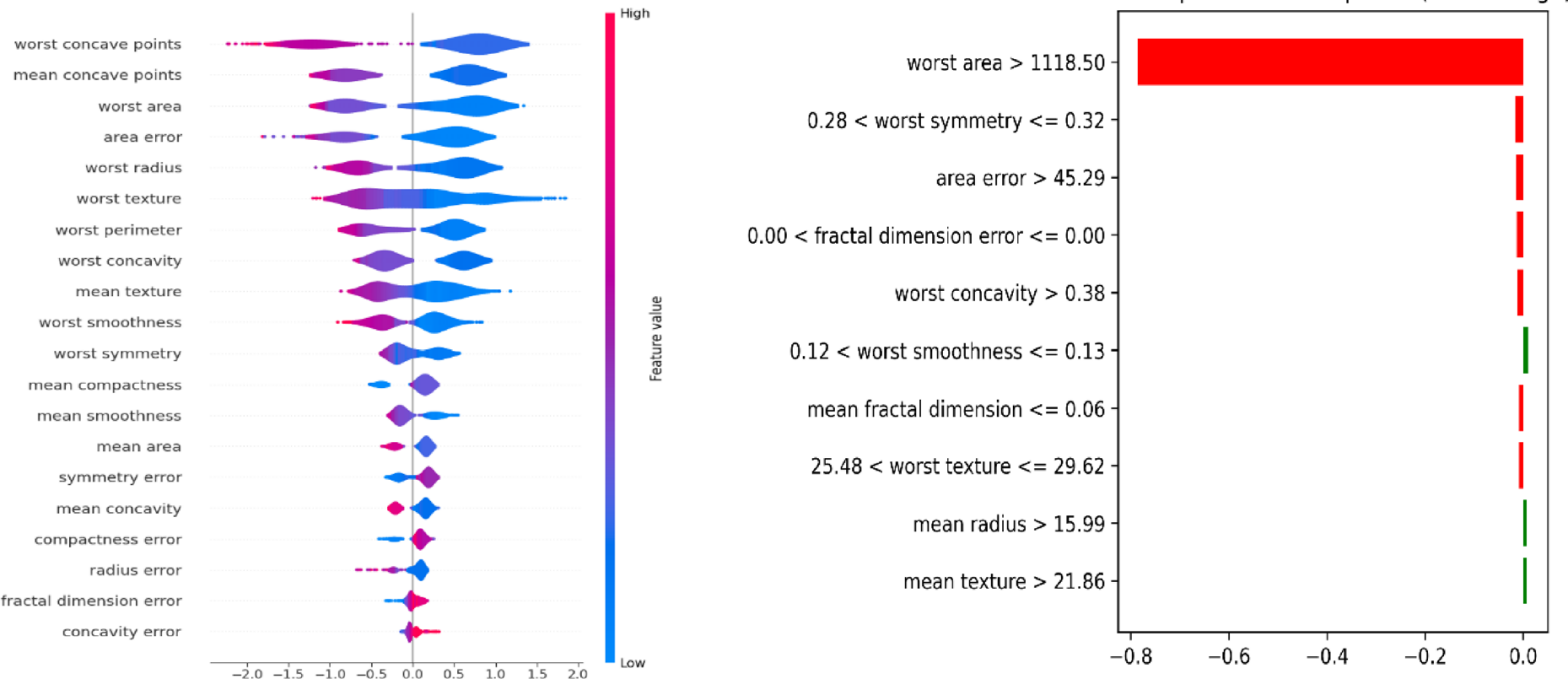
Impact of Model in SHAP and LIME.

Additionally, the well-liked method for providing a local and interpretable explanation for any machine learning model’s predictions is called Local Interpretable Model-agnostic Explanations (LIME)^14^. This is especially helpful when complicated models such as ensemble methods are involved, and it is necessary to comprehend how a model came to a specific conclusion. This model is mainly used in applications related to breast cancer, where the input data may be dynamic, diversified, and confront various difficulties. guarantees that this model is not unduly sensitive to small input changes and generalizes well. We employ data augmentation, adversarial training, and hybrid techniques to address input uncertainties to enhance resilience. The total dependability and credibility of our built system are influenced by both robustness and explainability. In terms of explainability, our model can aid in problem diagnosis and comprehension of the various ways the model operates under multiple circumstances, assisting in detecting and mitigating potential vulnerabilities. However, this model is more likely to instill trust due to its robustness and ability to function well under various circumstances. In essence, a new AI method is proposed with proof of both robustness and explainability. This comprises assessments using reference BC datasets to gauge the model performance in various scenarios and data metrics to reveal how our model makes decisions. Our model architecture and performance algorithms are shown in Fig. 3. The decision-making process helps clarify the model architecture.

### Future directions and limitations

In addition to the above contributions, many other aspects of this study have the potential to be advanced in some exciting ways. Therefore, this section suggests and discusses some aspects of future research. Although SeQTLBOGA has several benefits for diagnosing breast cancer, there are also some possible disadvantages, especially with regard to feature subset variability, dataset size, and overfitting concerns. Large, varied datasets are essential for the model’s success; smaller or unbalanced datasets may result in less-than-ideal feature selection and less generalizability. Another issue is overfitting, which occurs when high-dimensional feature spaces are used because the quantum-inspired optimization process may over-specialize to training data noise. Inconsistent clinical interpretations may also be introduced by variations in chosen feature subsets among runs or datasets, which could erode confidence in the model’s predictions. Future studies could investigate hybrid AI models that combine deep learning with quantum evolutionary algorithms to address these problems. These models would combine the representational strength of neural networks with the reliable search capabilities of SeQTLBOGA. Examining multi-objective optimization techniques that strike a compromise between feature selection effectiveness and classification accuracy is another exciting avenue to pursue. This will guarantee that the model retains good performance while reducing redundancy. Additionally, by utilizing pre-trained feature selection paradigms, transfer learning frameworks can expand the applicability of SeQTLBOGA to other medical classification issues. These developments could improve the model’s therapeutic usefulness, scalability, and dependability in a range of healthcare situations. In the updated manuscript, we also referenced a study on medical AI applications that used quantum evolutionary optimization. Overfitting, in which models learn noise rather than generalizable patterns, is made worse by small datasets. This results in inflated training accuracy but subpar test performance. Rotation gate learning rates are an example of a quantum metaheuristic with a high parameter complexity that may overfit to training data. Rankings of features’ importance are inconsistent. irrelevant feature selection, like giving noise precedence over biomarkers with clinical validation. Due to their extreme sensitivity to initialization, quantum algorithms generate feature subsets that vary from run to run. Clinical adoption is made more difficult by this instability since inconsistent biomarkers erode diagnostic confidence. For automated feature extraction, integrate deep learning architectures with quantum feature selection in the future.

The significant advantage of our algorithm is that it selects the best relevant features and also removes inconsistent features that cause the degradation of the accuracy of the classification algorithms. This algorithm plays a significant role in the accurate classification of BC data sets that work as a single objective. The disadvantage of our method is that it does not take advantage of parallel concurrent processing of high-dimensional data. Moreover, current studies should also inquire about patients who have completed treatment on what they believe to be the most crucial aspect in determining treatment release. We believe that the proposed system would be beneficial to doctors in making their final decisions on their patients. Therefore, our technique has a significant advantage in correcting breast cancer classification to achieve accurate decisions compared to existing algorithms, and on-time and accurate treatment may be provided to the patients. In future studies, we will use high-dimensional multi-class classification data to study breast cancer diseases that affect the performance of multi-classification methods.

## Experimental study and analysis

### Datasets description

Breast cancer is brought on by breast tumors, or abnormally developing cells in the breast tissue. The two BC dataset types, WBCD (Wisconsin Breast Cancer Database) and Wisconsin Diagnostic Breast Cancer (WDBC)^44^, obtained from human breast tissues, are categorization datasets that document breast cancer case measurements. Digital pictures of the breast tissues of the patients were used to compile 569 cases of the WDBC cancer dataset. The features mean, standard error, and worst or largest (mean of the three most significant values) were computed for each image, resulting in thirty features excluding id and label. It is split up into two categories. The WDBC dataset^45^ has 212 (37.2%) cases classified as malignant tumors and 357 (62.8%) cases classified as benign tumors. Only 699 suitable samples remained, and they were the only ones evaluated. The collection contains 65.5% (458) benign samples and 34.5% (241) cancer samples. Nine attributes defining the BC features are collected for each case sample. Data from 699 patients’ digital breast images are stored in WBCD. In each patient sample, 9 tumor traits are displayed^46^. 699 instances total from patient needle aspirates are included in the collection; 458 of these cases belong to the benign class, and the remaining 241 cases to the malignant class. Nine qualities are linked to every record in the database.

### Experimental setup

We have implemented our SeQTLBOGA approach, which employs EAs directly, in MATLAB R2016a on Windows 10 with an Intel^®^Core™ i7 processor and 16 GB of RAM. We used the Wisconsin Breast Cancer Databases utilizing this approach. The usual parameter settings were used in this paper to ensure a fair comparison. The following is the configuration for the SeQTLBOGA detail parameter.

#### Parameter configurations

For the EAs approached, primary parameters were established. These are the number of learners (population size) and the number of iterations is assigned the values 30 and 100, respectively. As a result, all EA algorithms, kept the population size and number of iterations constant. In this investigation, the number of iterations, population size, crossover probability, and mutation probability were the four primary parameters that is established for the GA technique corresponding values are 100, 30, 0.7, and 0.4. Quantum Rotation Angle ($\:\theta\:$) is $\:0.01\:\pi\:.$ Learning Rate set as 0.01, tournament size set as 3. Inertia Weight (w) = 0.4, Cognitive Coefficient (c_1), and Social Coefficient (c_2) set as 1.4; in DE the value of differential weight (F) set as 0.5.

### Performance measures

Depending on the particular application at hand, a variety of measures can be used to assess the EA algorithms. Researchers frequently use performance indicators like sensitivity and specificity to evaluate systems. In this part, we establish a set of performance indicators that we use to assess how well our technique is working. The performance metrics, such as accuracy, sensitivity, specificity, F-measure, and area under the curve (AUC), are shown as follows:

$$\:Accuracy\:=\frac{TN+TP}{TP+TN+FP+FN}\,Sensitivity=\:Recall\:\left(Re\right)=\frac{TP}{TP+FN}\,Precision\:\left(Pr\right)=\frac{TP}{TP+FP}$$

$$\:{F}_{score}=\frac{2*Pr*Re}{Pr+Re}\,AUC\:=\:\frac{(Sensitivity+\:Specificity)}{2}\,Specificity=\:\:\frac{TN}{FP+TN}$$

Additionally, True Positive Rate (TPR =$\:\frac{TP}{P}$), True Negative Rate (TNR = $\:\frac{TN}{N}$), False Positive Rate (FPR =$\:\frac{FP}{FP+FN})$, and False Negative Rate ( FNR = $\:\frac{FN}{TP+FN}$ ) are used to examine the proposed approach. Here, TP is the number of true positives; FN, the number of false negatives; TN, the number of true negatives; FP, the number of false positives.

### Results analysis and discussion

Our method is evaluated on WBCD and WDBC in this section. The proposed method applied with SVM training is compared to different up-to-date existing methodologies for detecting cancers by the expert system, including GA, DE, CS, PSO, and GOA. Results show that the proposed approach outperforms other EAs that are selected from up-to-date, relevant research found on used WBCD data using various modeling tools in previously referred literature^47^. All of the results presented are the averages of ten runs.

#### Comparison of feature selection methods using SVM

In situations where a complete evaluation appears unfeasible, the advantage of EAs like GA, DE, CS, PSO, and GOA is that they make it easier to select the best feature sets that promise improved performance. It chooses from two datasets the appropriate features. SeQTLBOGA, in conjunction with various evolutionary approaches and SVM learning variations, helps with classification optimization problems.

This section displays the results obtained from running our system on the WBCD and WDBC datasets. As a result, Table 1 displays the classification results of our system for WBCD and WDBC datasets, taking into account different setups of SVM classifiers and EAs-based feature selection techniques. This section presents the results of the evaluation of our method using five traditional EA-based studies as a comparison. Based on average metrics from ten runs of 10-fold cross-validation, these results are presented.

**Table 1:** Average performance in the wrapper methods of the two datasets.

WBCD						WDBC					
Classifiers	GA	PSO	CS	GOA	Proposed	Measure	GA	PSO	CS	GOA	Proposed
SVM-R	88.47	85.54	83.14	86.66	96.11	Accuracy	83.29	81.91	81.36	84.94	92.91
	80.62	81.69	84.98	84.45	92.62	Sensitivity	81.00	78.24	79.73	80.01	92.12
	83.55	82.04	81.68	83.89	93.54	Specificity	80.38	82.53	80.19	81.43	93.65
	81.23	80.64	83.44	82.81	92.11	F-measure	83.96	85.34	82.45	82.97	91.02
SVM-P	82.38	79.75	78.68	81.15	89.62	Accuracy	77.63	74.88	74.83	74.88	90.87
	80.22	78.01	79.37	79.01	82.62	Sensitivity	73.47	77.06	77.04	77.04	87.04
	77.81	76.05	77.16	75.11	80.65	Specificity	76.62	77.39	73.38	75.57	79.39
	79.65	76.72	77.88	78.45	81.06	F-measure	79.38	81.02	80.91	77.87	82.79
SVM-L	80.65	78.57	77.57	76.57	82.67	Accuracy	80.43	75.95	80.45	83.31	88.72
	73.04	77.68	76.53	72.4	83.21	Sensitivity	79.44	76.16	77.14	74.85	79.95
	70.84	72.49	75.45	71.02	81.81	Specificity	74.44	75.77	78.53	77.43	78.03
	71.07	72.91	75.62	77.98	85.35	F-measure	74.23	72.95	75.15	74.03	78.15

SVM-R classifier outperformed the other SVM classifier, based on Table 1 results. Additionally, proposed approach utilizing SVM-R performed better than the other methods, yielding outstanding outcomes when taking into consideration percentages for accuracy, sensitivity, F-measure, and specificity. Table 1 illustrates the efficacy of several wrapper techniques such as Genetic Algorithm, Particle Swarm Optimisation, Cuckoo Search, Grasshopper Optimisation Algorithm, and proposed method when utilised with SVM classifiers employing various kernels (RBF), Polynomial, and Linear on the WBCD and WDBC datasets. Our technique consistently surpasses GA, PSO, CS, and GOA across all SVM kernels. The SVM-RBF attains the best accuracy as 96.11%, sensitivity as 92.62%, specificity as 93.54%, and F-measure as 92.11% in WBCD, indicating exceptional discriminative capability in feature selection. Likewise, for SVM-Polynomial, our technique achieves an accuracy of 89.62%, markedly surpassing the next-best GA at 82.38%, while preserving balanced sensitivity as 82.62% and specificity as 80.65%. In the instance of SVM-Linear, the suggested methodology achieves an accuracy of 82.67%, although with a narrower margin, indicating that non-linear kernels (RBF, Polynomial) derive greater advantages from sophisticated feature selection.

The suggested method demonstrates superior performance, especially with SVM-RBF, with 92.91% accuracy, 92.12% sensitivity, and 93.65% specificity, signifying its resilience in managing WDBC data. The SVM-Polynomial achieves an accuracy of 90.87%, much above that of conventional optimisers GA as 77.63%, PSO as 74.88%, whilst the SVM-Linear demonstrates an accuracy of 88.72%, further affirming its generalisation capacity. The F-measure consistently exhibits a high value, reflecting a robust balance between precision and recall. Classical optimizers such as GA, PSO, CS, and GOA demonstrate modest efficacy, with GA typically outperforming the others, indicating that evolutionary algorithms are more consistent than swarm-based techniques like PSO, GOA. Non-linear SVM kernels (RBF, Polynomial) derive the greatest advantage from the suggested feature selection, presumably due to their superior management of intricate feature interactions.

Table 2 presents a comparison between the proposed method and the current SVM-R technique with respect to training and testing accuracy and estimated run time. Based on ten rounds of tests using both datasets, these conclusions have been drawn. Based on the statistics, it can be concluded that the proposed method consistently yields more reliable and consistent results than its five rivals.

**Fig. 4:**
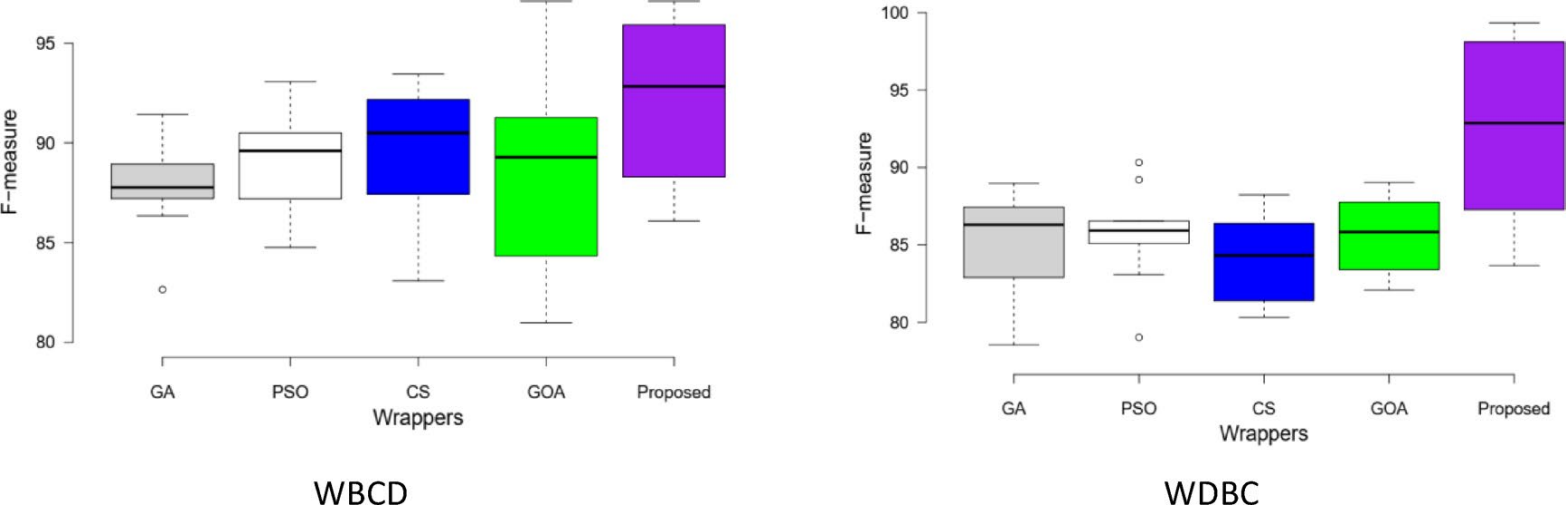
Box-plot of wrappers of F-measure rate on both datasets.

#### Comparative studies in terms of performance

In this section, our method outcomes and debates are shown alongside five alternative wrapper strategies that use an SVM-R classifier. Table 2 shows that the training accuracy rate for the proposed system is 98.95, and the WBCD execution time is 877.98 (in seconds). Additional analysis has been done on the WDBC dataset 97.43, as Table 2 displays the training accuracy rate of 544.89 (in seconds). It provides an overview of the BC datasets-focused performance analysis. The results of our strategy and additional methods are displayed in the table, accounting for factors. As seen by Fig. 4, the F-measure rate, we can demonstrate that our approach outperforms existing state-of-the-art EAs in terms of performance; it achieves an optimal sensitivity rate on the dataset. When compared to previous strategies, the results show that our technique produces better operational results in terms of classification performance. As demonstrated in Tables 1 and 2. Furthermore, as compared to the previous strategies used, the proposed strategy greatly reduces training and testing times.

**Table 2:** Evaluation of BC databases on different eas.

Data	Methods	Parameters		Train Accuracy	Test Accuracy	Runtime(secs)
		σ	c			
WBCD	Single SVM	0.993	5.342	98.87	97.65	1022.43
	DE-SVM	0.892	4.987	98.32	98.01	995.45
	GA-SVM	0.873	4.556	98.23	98.12	890.32
	CS-SVM	0.786	3.654	96.34	95.99	1011.34
	GOA-SVM	0.432	3.112	92.54	91.57	889.04
	SeQTLBOGA	0.065	1.443	98.95	98.34	877.98
WDBC	Single SVM	0.889	5.453	89.32	88.11	1088.43
	DE-SVM	0.765	3.554	87.45	85.43	1011.43
	GA-SVM	0.544	3.654	88.11	86.98	899.90
	CS-SVM	0.435	3.987	89.23	87.22	880.54
	GOA-SVM	0.323	1.342	89.32	88.42	911.32
	SeQTLBOGA	0.034	1.123	97.43	94.19	544.89

The performance of the proposed method is compared over time to different wrapping techniques for different types of malignancies using simulations. Table 3 presents an analysis of the results of comparing the suggested method with five conventional studies, as well as a comparison with several state-of-the-art methodologies. The comparison was initially done for datasets from UCI. Table 3 compares the suggested model for classification on BC datasets with the comparative analysis of EAs such as GA, DE, CS, PSO, and GOA.

**Table 3:** Comparative analysis of the experimental performance on two datasets.

	WBCD							WDBC					
K-Fold	Proposed	GA	PSO	DE	CS	GOA	Measure	Proposed	GA	PSO	DE	CS	GOA
2	99.43	89.34	87.42	88.67	89.21	87.02	Sensitivity	93.45	92.98	89.03	88.56	87.23	88.34
4	99.00	90.22	84.43	86.34	82.02	81.76		92.99	90.76	89.09	81.89	83.76	85.23
6	98.31	92.43	95.03	94.77	96.02	95.95		90.12	87.43	86.46	85.23	82.45	82.98
8	98.62	95.54	86.78	91.43	90.65	92.65		91.78	90.34	88.21	83.04	80.92	85.23
10	99.11	94.54	95.23	94.79	95.98	96.03		94.23	90.78	89.23	85.43	87.23	82.98
2	98.54	97.45	96.34	95.65	94.55	97.23	Specificity	88.67	88.32	84.5	82.56	80.67	85.98
4	96.47	95.33	94.02	94.00	94.67	95.43		86.32	80.12	81.23	80.43	82.43	83.64
6	97.89	96.98	95.88	96.03	98.45	98.01		87.12	81.32	86.02	82.32	81.32	82.54
8	96.66	91.43	90.87	89.03	93.43	88.04		81.32	8027	81.87	82.87	81.09	78.67
10	97.21	96.03	95.55	94.78	93.09	94.67		77.23	78.54	79.76	75.27	76.87	78.99
2	344.34	388.45	390.76	498.43	789.34	1000.4	Etr(sec)	123.54	166.54	187.76	200.43	189.34	212.56
4	423.87	546.23	672.54	657.55	711.45	690.54		156.76	154.66	298.22	156.67	187.21	211.05
6	576.54	723.45	833.65	743.37	900.23	892.45		232.44	254.23	322.49	233.44	298.23	311.43
8	981.32	1111.45	1453.54	1004.54	966.45	897.56		433.54	456.44	533.65	675.33	644.34	634.43
10	865.56	980.34	879.34	1023.87	1117.8	1000.4		511.34	522.23	799.12	583.66	546.71	567.11
2	1189.04	1345.22	1211.56	1342.65	1411.23	1244.65	Ete(sec)	709.02	1008.4	988.43	899.34	923.45	834.6
4	1243.43	1232.43	1304.5	1299.45	1344.5	1444.45		699.34	934.54	899.34	922.45	899.34	902.54
6	1356.45	1511.43	1623.56	1322.65	1228.56	1235.65		733.45	965.45	877.65	875.45	788.45	899.34
8	1427.80	1511.34	1345.23	1123.43	1228.45	1311.45		945.56	1324.43	1022.5	933.5	1056.45	1077.98
10	1132.98	1332.57	1244.47	1542.25	1622.31	1445.65		1070.5	1277.5	1321.65	1225.6	1355.4	1233.6

Furthermore, it is evident that our strategy performs better than the feature subset in both datasets based on the actual results obtained by testing the suggested technique on the dataset. Based on these data, our approach performs better than other state-of-the-art methods, as shown by all the evaluation indicators listed in Table 4.

**Table 4:** Comparative analysis of proposed in datasets.

Method	Dataset	Accuracy
^48^	WBCD	96.56
^49^	WBCD	91.15
50	WDBC	95.17
^51^	WDBC	96.49
^51^	WDBC	94.74
**our**	WDBC	97.43

As shown in Table 4, the proposed approach has achieved the highest accuracy compared to other recent techniques, such as^47–49^ In addition to its superior accuracy, the proposed technique also significantly outperforms other prevailing state-of-the-art techniques in terms of accuracy.

**Fig. 5:**
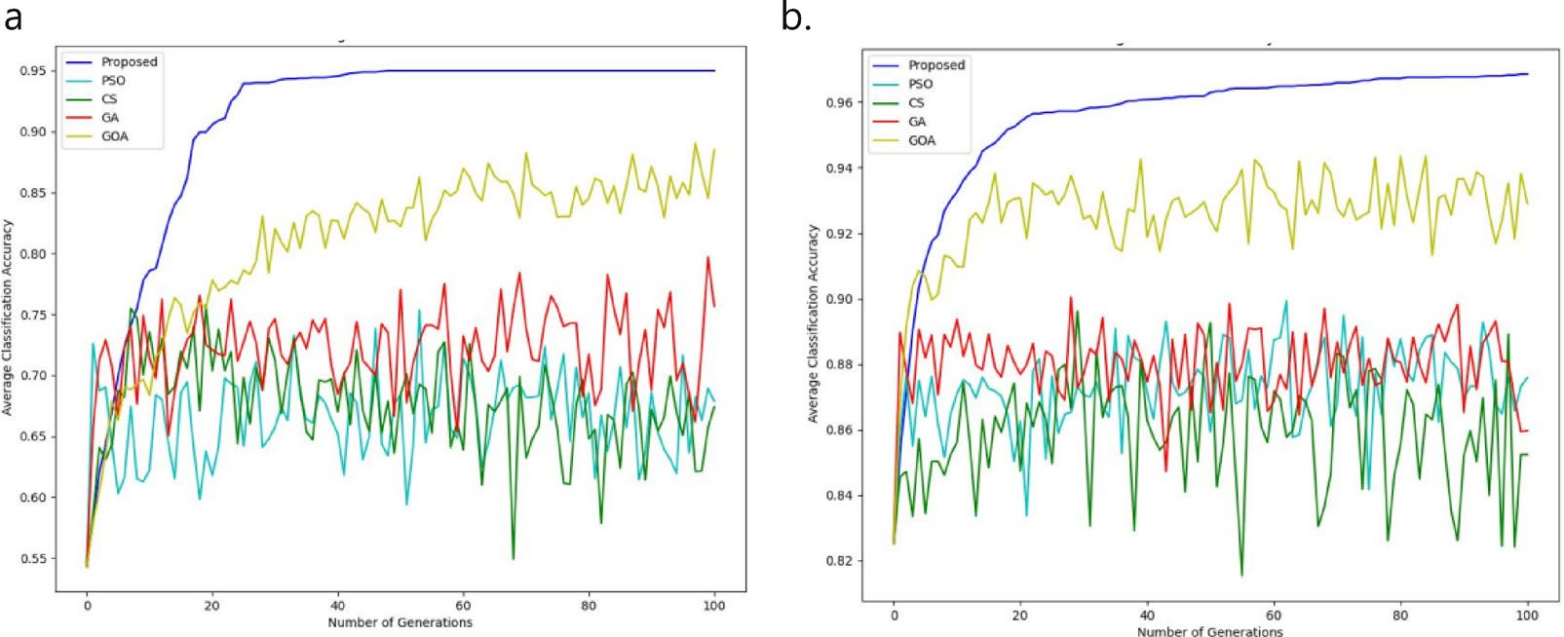
Convergence graph on (**a**) WBCD. (**b**) WDBC.

Figure 5 shows the convergence graph on two datasets based on WBCD and WDBC, respectively. From graphs, we can see that our proposed method performs best regarding classification accuracy compared to other nature-inspired algorithms.

**Table 5:** AUROC and AUPR values dataset.

Datasets	Measures	GA	PSO	DE	CS	GOA	Proposed
WDBC	AUROC	96.99	97.43	89.34	98.32	94.23	99.06
	AUPR	97.66	98.22	89.98	98.03	96.47	99.06
WBCD	AUROC	98.23	97.44	95.03	97.34	98.90	99.11
	AUPR	97.34	95.22	94.34	94.88	98.91	99.12

Classifier performance is measured using AUPR (Area under Precision-Recall) Curve values ranging from 0 to 1, with a perfect classifier reaching an AUPR score of 1. Table 5 shows the AUROC and AUPR values, suggesting that the proposed technique outperforms other current models in identifying disease across the dataset used. In contrast, all of the techniques used have a very good fit. In Table 6, Central Nervous System (CNS)^52^ Data shows the comparative study by our wrapper method and another wrapper method in terms of four measures, like accuracy, F-measure, sensitivity, and specificity.

**Table 6:** Comparative analysis of four measures on the CNS dataset.

EAs	Performance	SVM-L	SVM-*P*	SVM-*R*
GA	Accuracy	83.52	81.25	86.32
	Sensitivity	82.25	80.25	84.63
	Specificity	78.63	79.36	85.6
	F-measure	84.25	78.31	84.87
PSO	Accuracy	85.67	83.54	81.63
	Sensitivity	82.63	81.74	80.52
	Specificity	79.34	79.86	79.38
	F-measure	80.52	76.58	77.94
CS	Accuracy	75.36	74.22	76.32
	Sensitivity	74.25	71.02	70.05
	Specificity	77.89	75.23	71.95
	F-measure	72.84	73.65	74.25
GOA	Accuracy	78.25	73.56	74.85
	Sensitivity	77.84	73.89	73.45
	Specificity	76.52	75.89	79.65
	F-measure	77.25	76.84	77.06
Our	Accuracy	88.25	87.62	85.32
	Sensitivity	87.67	85.66	84.79
	Specificity	84.63	86.07	85.97
	F-measure	84.25	83.96	82.78

#### Statistical results

One of the most popular statistical techniques for ranking algorithm performance is the Friedman test^53,54^. Whether looks for any discernible differences between the outcomes of several algorithms. Its foundation is the null hypothesis, which states that there are no differences in how the algorithms are presented. Based on the following factors, the algorithm that performs the best gets the lowest rank, while the algorithm that performs the worst gets the highest rank (see Table 7).

1. The observed accuracy value for each algorithm and dataset pair.
2. For each BC dataset, rank values from 1 (best result) to 5 (worst result).
3. For each method, average the ranks obtained in the BC datasets to achieve the final rank.

**Table 7:** Summary of statistical results.

Algorithms	Ranking
GA	2.25
PSO	3.875
CS	5
GOA	2.875
Proposed	1

The tests are applied to the accuracy obtained from the BC data sets. Table 7 displays Friedman’s statistic, which determines the level of responsibility based on the average rank that each method achieved on two datasets. Based on the computed results, the calculated Friedman statistic is 14.95, and the test p-value was 0.004806 at the 5% significance level. As a result, the null hypothesis is disproved, indicating variations in the methods’ performances. Using Li and Holm’s process, the post-hoc method for pairwise comparisons of metaheuristic methods further validates the suggested method. P-values that were determined by utilizing post hoc techniques on the Friedman procedure findings are displayed in Table 8.

**Table 8:** Post hoc comparison table for $\:\alpha\:$ = 0.05.

i	Algorithms	z = (R0 − Ri)/SE	*p*	Holm Hochberg	Li
4	GA	3.577709	0.000347	0.0125	0.03876
3	PSO	2.571478	0.010127	0.016667	0.03876
2	CS	1.677051	0.093533	0.025	0.03876
1	GOA	1.118034	0.263552	0.05	0.05

**Fig. 6:**
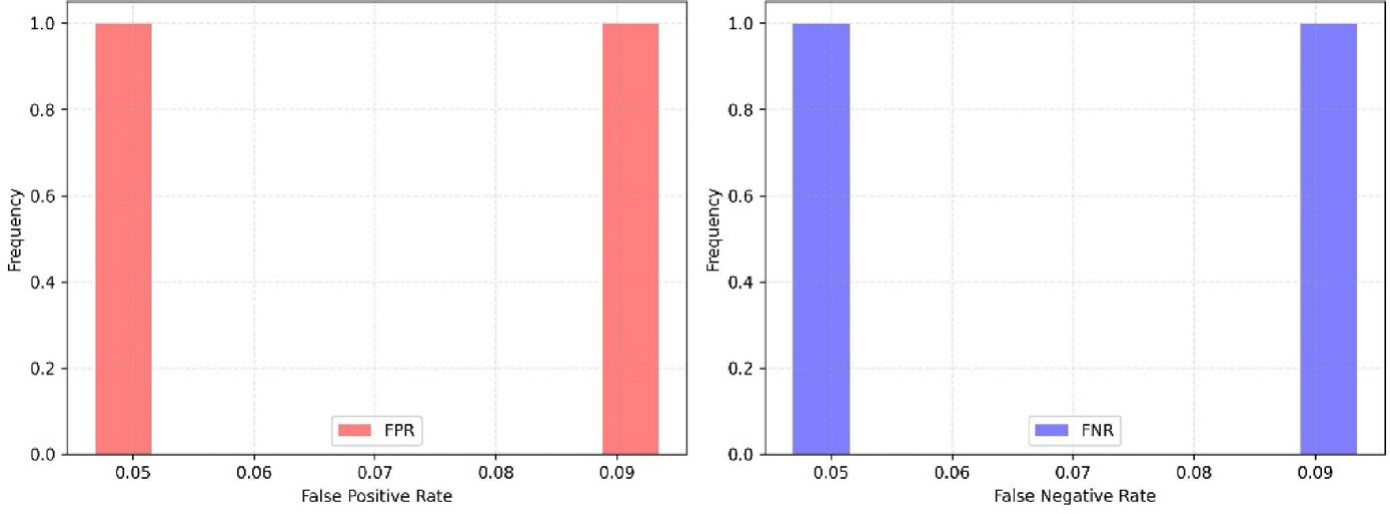
Histograms of false positive and false negative rates.

In order to comprehend model performance and potential biases, error analysis in breast cancer datasets entails assessing false positive (FP) and false negative (FN) rates. False negatives happen when a cancer case is overlooked, possibly postponing treatment, whereas false positives happen when a benign case is mistakenly identified as malignant, resulting in needless operations. The goal of bias detection is to determine if these mistakes are consistently linked to particular characteristics, as seen in Fig. 6.

## Conclusion

Breast cancer is a prevalent disease that affects women globally. However, the number of deaths from breast cancer has decreased by the early detection of the disease through the application of medical evolution techniques. Particularly in breast cancer prediction, our novel development based on a self-adaptive quantum evolutionary algorithm known as SeQTLBOGA is vibrant for enhancing the quality of life for millions of patients. Our method has two main benefits. First, using fewer features can build a diagnostic model that is easy to understand. The other is that optimizing the SVM model parameters can produce the best prediction model. Specifically, using a series of experiments with EAs on both BC databases, we show that our method selects the most valuable features and maximizes speed. The experimental results show that our method produces more accurate results than regular EAs when EAs are combined with SVM. Our method performs best in BC datasets regarding ROC area, F-measure, accuracy, sensitivity, and specificity. These findings demonstrate that our technology can be used as a practical replacement for the existing techniques for diagnosing breast cancer. We may apply our SeQTLBOGA with the SVM kernel to predict the prognosis of additional deadly illnesses. It will consequently be a lifeline for patients in terms of the clinical management of cancer and other life-threatening conditions and their prognosis. This algorithm can be utilized as a diagnostic tool in BC research.

## Data Availability

The datasets used during the current study are publically available in the Wisconsin Diagnostic Breast Cancer datasets. The data generated during the current study available from the corresponding author on reasonable request.
